# Epidermal–dermal coupled spheroids are important for tissue pattern regeneration in reconstituted skin explant cultures

**DOI:** 10.1038/s41536-023-00340-0

**Published:** 2023-11-23

**Authors:** Mingxing Lei, Jingwei Jiang, Mengyue Wang, Wang Wu, Jinwei Zhang, Wanqian Liu, Wei Zhou, Yung-Chih Lai, Ting-Xin Jiang, Randall B. Widelitz, Hans I-Chen Harn, Li Yang, Cheng-Ming Chuong

**Affiliations:** 1 Key Laboratory of Biorheological Science and Technology of Ministry of Education & 111 Project Laboratory of Biomechanics and Tissue Repair, College of Bioengineering, Chongqing University Chongqing, 400044 China; 2 Integrative Stem Cell Center, China Medical University Hospital, China Medical University, Taichung 40402 Taiwan; 3 Chongqing Key Laboratory of Translational Research for Cancer Metastasis and Individualized Treatment, Chongqing University Cancer Hospital, Chongqing, 400030 China; 4 Department of Pathology, Keck School of Medicine, University of Southern California, Los Angeles, CA 90033 USA

**Keywords:** Skin stem cells, Apicobasal polarity

## Abstract

Tissue patterning is critical for the development and regeneration of organs. To advance the use of engineered reconstituted skin organs, we study cardinal features important for tissue patterning and hair regeneration. We find they spontaneously form spheroid configurations, with polarized epidermal cells coupled with dermal cells through a newly formed basement membrane. Functionally, the spheroid becomes competent morphogenetic units (CMU) that promote regeneration of tissue patterns. The emergence of new cell types and molecular interactions during CMU formation was analyzed using scRNA-sequencing. Surprisingly, in newborn skin explants, IFNr signaling can induce apical-basal polarity in epidermal cell aggregates. Dermal-Tgfb induces basement membrane formation. Meanwhile, VEGF signaling mediates dermal cell attachment to the epidermal cyst shell, thus forming a CMU. Adult mouse and human fetal scalp cells fail to form a CMU but can be restored by adding IFNr or VEGF to achieve hair regeneration. We find different multi-cellular configurations and molecular pathways are used to achieve morphogenetic competence in developing skin, wound-induced hair neogenesis, and reconstituted explant cultures. Thus, multiple paths can be used to achieve tissue patterning. These insights encourage more studies of “in vitro morphogenesis” which may provide novel strategies to enhance regeneration.

## Introduction

Tissue patterning is a fundamental process that occurs in developing skin and wound-induced hair neogenesis^1^. Recent work shows tissue patterns can also be produced in reconstituted skin explants. Reconstituted skin explants are three-dimensional (3D) cultures of stem cells or progenitor cells that can recapitulate the morphologies and functions of multiple physiologically developed epithelial organs^2^, providing a robust platform to model tissue morphogenesis, regeneration, repair, disease, and drug screening^3^. Here, we explore how the skin progenitors organize themselves for further morphogenesis. The chemical and physical environments of skin explants undergoing morphogenesis are constrained and differ from those found in developing skin and adult wounds^4^. The cellular states and their environments in these different scenarios are very different and dissimilar molecular pathways are used to attain the final tissue patterns^5–7^.

We hypothesize alternative morphogenetic pathways may operate in the skin explant which may be parallel, yet not identical, to those used in developing skin. Here we ask what cell types and molecular signaling pathways are used in reconstituted skin explants to establish morphogenetic competence required for regeneration and how they differ from those used in development and wound regeneration. We aspire to identify key “morphogenesis markers” that can be used as landmarks to predict the regenerative behavior of reconstituted skin explants (markers at the tissue level) and guide the designing of culture protocols, just like molecular markers have been useful in predicting stem cell behaviors (markers at the cell level).

For the skin, pioneering skin reconstitution research was done by Yuspa’s group in the 1990s. They mixed skin progenitor cells in a small chamber that was built on the back of nude or SCID mice. This produced good hair growth after the chamber was removed^8^. Subsequently, scientists have tried different variations. For example, in the patch assay, progenitor cells are injected into the dermis, and big cysts with hairs pointing inward form^9^. Using an extracellular matrix and defined soluble factors, Chacón-Martínez et al. 3-dimensionally cultured hair follicle stem cells that also form aggregates and regenerate hair follicles upon transplantation^10^. Cells obtained from different species (including humans) were grown in skin explant cultures using different culture conditions^11–13^. More recently, we were able to culture dissociated skin cells for about 10 days, making them multipotential and competent for self-organizing tissue patterns, then transplant them onto nude mice^14^. A major advance in the field is the generation of skin organoids using pluripotential embryonic stem cells or iPS from human^15,16^ or mouse^15,16^. It is also possible to generate epidermal stem cells from adult mice and keep them in long-term culture in 3D epidermal cyst morphology^17^. From different paths, these engineered skin products can promote the formation of functional skin^15,16,18^. Since different multi-cellular configurations and molecular pathways are used to achieve morphogenetic competence in developing skin, wound-induced hair neogenesis, and reconstituted explant cultures^14^, we need to learn more about how the morphogenetic processes progress in these reconstituted skin or organoids.

In our newborn reconstituted skin explants derived from newborn mouse cells, we found numerous spheroids, but not in those derived from adult mouse cells^14^. Reconstituted skin explant cultures derived from mouse and human pluripotent stem cells have been shown to form large cysts^15,16^. Yet many skin explants using cells from other sources do not regenerate hairs successfully or show only partial success, such as human scalp/foreskin cells^19^, or dermal papilla cells without stem-like epidermal keratinocytes^20^. Here, we clarify the terminology used in the explant cultures. A cyst represents a thin-walled hollow structure made of epithelial cells with a cavity containing air or liquid secretion, without dermal cells attached on the outside. Spheroid is a nearly round aggregate of cells that can be homotypic (made of the same kind of cells such as dermal cell spheroid) or heterotypic (at least two cell types, and they can mix evenly or form two subgroups in a certain topology). It is important to differentiate these in explant cultures, as their progression is significant for in vitro morphogenesis. In our explants cultures, the epidermal-dermal coupled spheroids represent an epidermal cyst with a basement membrane and a few layers of dermal cells attached on the outside. We hypothesize the emergence of epidermal-dermal coupled spheroid topology is a cardinal morphological feature indicative of the ability to undergo further morphogenesis and regenerate after grafting. Therefore, we name this epidermal-dermal coupled spheroid as a “competent morphogenetic unit” (CMU) to indicate this function.

To reach a higher resolution understanding, we apply single-cell RNA-sequencing (scRNA-seq) to analyze newborn mouse skin explants that regenerate and adult mouse skin explants that do not. We analyze dynamic changes and monitor cell clusters before and after CMU formation. We study intercellular communications in newborn skin explants at these stages using the CellChat algorithm^21^. By comparing the data from newborn and adult explants, we identify factors and cell communications that are lacking in the adult explants. By adding these factors back, we can rescue the regeneration ability of adult mouse explants. Applying similar explant culture protocols to human fetal scalp-induced cyst formation in culture and hair follicles after being transplanted to nude mouse skin.

## Results

### Transcriptome profiling during the formation of epidermal–dermal spheroids

**Fig. 1:**
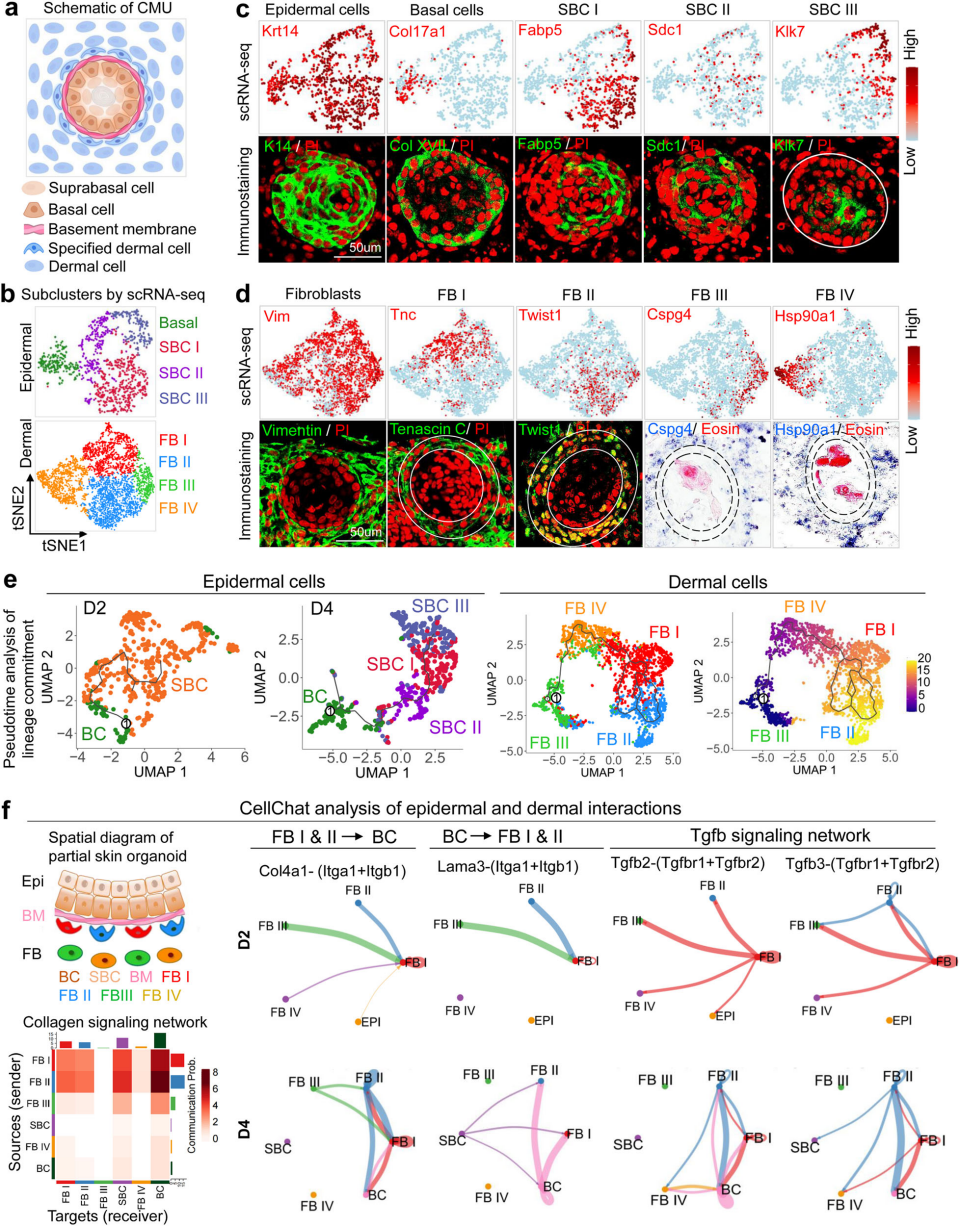
ScRNA-seq analysis reveals heterogeneity of newborn mouse epidermal and dermal cells during the formation of CMU. **a** Schematic drawing of a CMU. **b** Cell clustering of newborn mouse epidermal and dermal cells of combined D2 & D4 cultures. **c** TSNE plots and immunostaining for expression of exemplary genes in newborn mouse epidermal cells, basal cells, suprabasal cells I (SBC I), SBC II, and SBC III. **d** TSNE plots and immunostaining for expression of exemplary genes in newborn mouse dermal fibroblasts (FB), FB I, FB II, FB III, and FB IV. **e** Pseudotime analysis of lineage commitment of newborn mouse epidermal (D2 and D4) and dermal cells (D4). **f** CellChat analysis of newborn mouse epidermal and dermal interactions during CMU formation. False discovery rate < 0.05 and Log2 fold change > 1.

When grown as an explant culture, combined dissociated newborn mouse epidermal and dermal cells form a spheroid composed of 1–3 layers of dermal cells on the outside overlying an epidermal cell ring encircling keratin debris within the center (Fig. 1a). The spheroids either form a planarized skin or maintain a spheroid topology after transplantation onto the dorsum of nude mice (Supplementary Fig. 1a). K14 immunostaining shows that hair follicles can form either from the spheroids (15 ± 5%) or the later staged planarized skin (85 ± 5%) (Supplementary Fig. 1a). We previously demonstrated that the cysts coalesce to form a planarized skin in newborn mouse cell culture, whereas adult mouse cells grown in explant cultures form epidermal aggregates but do not form these epidermal-dermal coupled spheroids or planarized skin nor hair follicles^14^. Therefore, this finding suggests that the skin spheroid represents a unique morphological marker for morphogenesis in cultured reconstituted skin explants. By quantifying the cellular components at the middle cross-section level, we observe that there are 15–35 basal epidermal cells, 3–5 layers of suprabasal epidermal cells, a basement membrane, and 1–4 layers of dermal cells.

To examine the cellular and molecular components of these spheroids, we profiled 8558 single cells from D2 (day 2) and D4 (day 4) newborn mouse skin explant cultures using scRNA-seq (Supplementary Fig. 1b and Supplementary Table 1). T-distributed stochastic neighbor embedding (tSNE) plots of D2 and D4 newborn mouse skin explant cultures reveal 11 cell clusters, with epidermal cells and dermal fibroblasts as the two major sub-clusters (Fig. 1b). Since polarization occurs in epidermal cells (K14+), we isolated these cells and performed tSNE plot analysis, which reveals four main epidermal cell clusters (Fig. 1c) including basal cells (Col17a1+) and suprabasal cells I (SBC I, Fabp5+), SBC II (Sdc1+), and SBC III (Klk7+) (Fig. 1c and Supplementary Fig. 1c). ScRNA-seq analysis also reveals four dermal fibroblast groups including fibroblast I (FB I)–FB IV, which show high expression of representative genes such as Tnc, Twist1, Son, and Hsp90a1, respectively (Fig. 1d), as well as other highly expressed genes (Supplementary Fig. 1c). Pseudotime analysis by Uniform Manifold Approximation and Projection (UMAP) plots reveals that the basal layer can differentiate into the suprabasal layer at D2, which can further differentiate into another two suprabasal cell types at D4 (Fig. 1e). Pseudotime analysis also reveals that FB III can undergo lineage commitment to FB IV, FB I to FB II, with specific molecular identity in each cell cluster (Fig. 1e and Supplementary Fig. 1c).

### Epidermal–dermal coupling is required for the establishment of competent morphogenesis units

To examine whether epidermal cells interact with dermal cells to form functional CMUs, we isolated epidermal and dermal cells from the D2 and D4 newborn mouse skin explant cultures, in which six main clusters were characterized by tSNE plots, including four dermal fibroblast clusters (FBs I–IV) and two main epidermal clusters (BC & SBC), (Fig. 1f and Supplementary Table 2). We then used CellChat to study the communications between epidermal and dermal cells^21^. CellChat analysis of these six clusters identified FB I, FB II, and basal cells as the dominant communication “hubs” that secrete and receive signals (Supplementary Fig. 1d). FB I and FB II secrete the majority of Collagen whereas basal cells highly express Laminin. Collagen and Laminin are the top 2 secreted ECM outgoing signaling patterns (Supplementary Fig. 1d left). Basal epidermal cells can receive the vast majority of ECM signaling secreted by both dermal cells and epidermal cells (Supplementary Fig. 1d right). This implies that molecular interactions occur between epidermal and dermal cells.

The CellChat-inferred Collagen signaling pathway further confirms that FB I and FB II are the primary ligand sources, which act not only in an autocrine manner between dermal cells but can act even more strongly as paracrine signals from dermal to epidermal cells (Fig. 1f lower left). Indeed, CellChat identified ligand-receptor pairs including Collagen-Integrin as the most significant signaling contributors from FB I and FB II to basal cells (Fig. 1f). FB I- and FB II-expressed Collagen IV can be received by Integrins a1 and b1 in basal cells. Conversely, CellChat identified Laminin-Integrin as the major signaling molecule for communication from basal cells to FB I and FB II. Here, basal cells express Laminin signals that can be transmitted to FB I and II which also express Integrins a1 and b1. These laminin-integrin interactions might induce CMU formation. Interestingly, epithelial–mesenchymal interactions (EMI) are enhanced during the CMU formation process from D2 to D4, as indicated by the increased interactions of Collagen and Laminin at D4 (Supplementary Fig. 1e). CellChat analysis also shows that FB I and II secrete most of the signaling molecules, which can be received by Basal cells (Supplementary Fig. 1f). For example, Tgfb signaling pathway genes are highly expressed in FB I and FB II and can be received by Tgfbr1 and Tgfbr2 expressed by the basal cells (Fig. 1f right). Together, these data suggest that dermal cell-derived ECM signaling may regulate epidermal cell behavior, and vice versa during CMU formation.

### Tgfb and Gsk3 regulate ECM expression and basement membrane organization to control epidermal apical-basal polarity

**Fig. 2:**
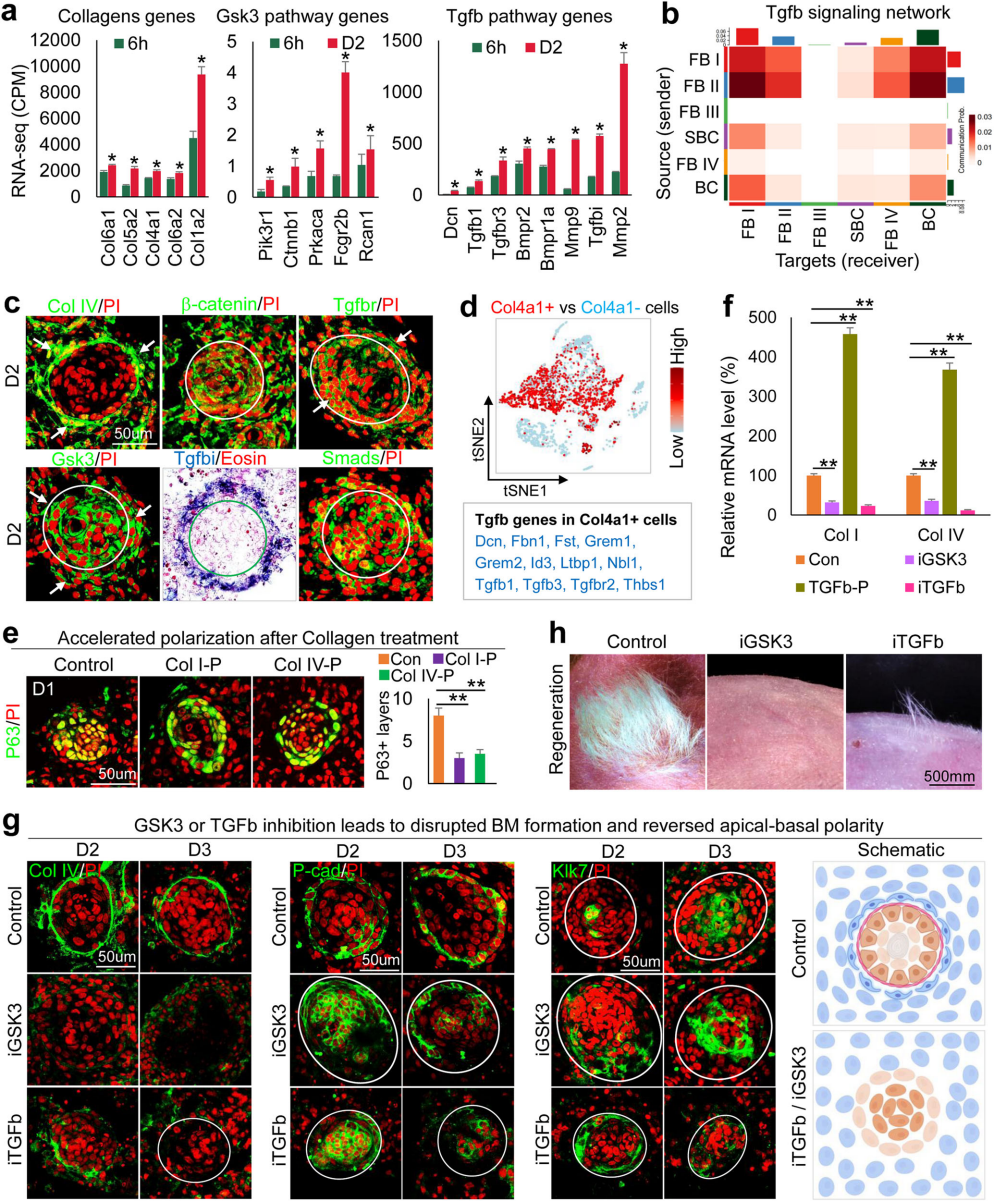
Tgfb–Gsk3 controls the polarization of epidermal cells in newborn mouse cell aggregates by modulating ECM polymerization and induces basement membrane formation. **a** RNA-seq analysis shows that ECM, Gsk3, and Tgfb pathway genes are upregulated from 6h to D2. *p < 0.05, *n* = 2. **b** Heatmap of senders and receivers of Tgfb signaling. **c** Immunostaining and in situ hybridization show gene expression in D2 culture. **d** Tgfb pathway genes are highly expressed in Col4a1+ vs. Col4a1- cells. **e** Immunostaining and quantification for P63 show accelerated polarization at D1 after the addition of Collagens I or IV at D0. **p < 0.01. *N* ≥ 3. **f** qRT-PCR analysis reveals Col1a1 and Col4a1 expression after inhibition of GSK3 (iGSK3) or iTGFb or the addition of TGFb recombinant protein (TGFb-P). **p < 0.01. *N* = 3. **g** Immunostaining of Collagen I, P-cadherin, and Klk7 expression after GSK3 or TGFb inhibition. **h** Hair regeneration with transplantation of iGSK3- or iTGFb-treated cultures.

Since the CMU is composed of three cellular components, we began to explore how the basement membrane formation influences epidermal apical–basal polarity. RNA-seq analysis of D2 (polarized state) and 6h (unpolarized state) newborn mouse skin explant samples revealed that the ECM gene ontology (GO) terms rank prominently (Supplementary Fig. 2a). ECM genes (e.g., Collagens) are significantly increased from 6h to D2 (Fig. 2a, Supplementary Fig. 2b, and Supplementary Table 2). Interestingly, we also observed that Gsk3 pathway-related genes are significantly increased from 6h to D2 by bulk RNA-seq analysis (Fig. 2a and Supplementary Fig. 2c). TSNE plots show that Gsk3b colocalizes with Col1a1 and Col4a1 in dermal cells but not in epidermal cells (Supplementary Fig. 2d), which is verified by immunostaining (Fig. 2c). Since Gsk3 is a Wnt signaling inhibitor, we checked b-catenin expression in D2 cultures and observed that it is not activated in specified dermal cells (Fig. 2c), indicating Gsk3 may function to repress Wnt signaling. To test the involvement of GSK3+ on collagen expression, we isolated Gsk3+ dermal cells and performed GO analyses, which revealed several ECM molecule terms including collagen that may act downstream of Gsk3 (Supplementary Fig. 2e).

To probe the upstream regulators of Gsk3 that modulate ECM expression, we analyzed our bulk RNA-seq data which reveals that Tgfb pathway genes are significantly increased from 6h to D2 in newborn mouse skin explant cultures (Fig. 2a). Using CellChat secreted signaling mode analysis, we observed that Tgfb signaling pathway genes are highly expressed in FB I and FB II (Fig. 2b), with multiple Tgfb ligand-receptor pairs contributing to the EMIs (Supplementary Fig. 2f). In situ hybridization and immunostaining showed that Tgfb pathway genes are expressed in the specified dermal cells and the basal epidermal cells (Fig. 2c). Since Col4a1 is highly expressed in FB I and FB II, we analyzed the gene expression profile in Col4a1+ cells versus Col4a1- cells and observed that Tgfb pathway genes are indeed highly enriched in Col4a1+ cells (Fig. 2d). We then evaluated the properties of the FB II cell cluster which is the biggest source of Tgfb signaling (Fig. 2b). Four sub-types of fibroblast cells (FB II-A, B, C, D) can be further identified when the FB II cluster was subjected to a deeper tSNE plot analysis. FB II-A shows high expression of dermal papilla marker genes including Bmp4, Bmp7, Lef1, and Ltbp1 (Supplementary Fig. 2g). These data suggest that the Tgfb+ FB II-A cell population contains the cells that are potentially the competent cells for prospective regeneration (e.g., the pre-dermal condensate cells) during CMU formation.

Since Collagens I and IV are known basement membrane components, we tested their function by adding Collagen I or IV recombinant protein to the skin explant cultures. Exogeneous Collagen proteins can be incorporated into the basement membrane region and dermal cells. This leads to accelerated basement membrane formation and mature polymerized collagen fibers in the collagen-treated groups compared to the control group which shows immature collagen expression and a relatively incomplete basement membrane (Supplementary Fig. 2h). The addition of Collagen I or IV did not significantly change the aggregate size, but dramatically increased the dermal cell layers surrounding the aggregate (Supplementary Fig. 2i). As a result, the polarization process is accelerated in the Collagen-treated groups compared to the control at D1, as shown by P63 immunostaining (Fig. 2e). This suggests that dermal cell-secreted Collagens promote epidermal cell polarization in newborn mouse skin explant culture.

Since the expression of Gsk3 pathway genes is increased from 6h to D2 and Gsk3 colocalizes with Col1a1 and Col4a1 in dermal cells (Fig. 2a and Supplementary Fig. 2c and d), we tested if Gsk3 signaling regulates Collagen expression by treating D0 cultures with a Gsk3 inhibitor, Chir99021. We observed that Col1a1 and Col4a1 mRNA and protein levels are significantly decreased at D2 compared to controls as shown by qRT-PCR and immunostaining, respectively (Fig. 2f, g and Supplementary Fig. 2k). Particularly, while GSK3 inhibition does not influence aggregate size (Supplementary Fig. 2i), it decreased Collagen I and IV expression in the specified dermal cells (Fig. 2g left and Supplementary Fig. 2k). This resulted in incomplete basement membrane formation (Fig. 2g) and decreased b1-integrin expression in the basal epidermal aggregate compared to the control (Supplementary Fig. 2m). Without the dermal ECM and basement membrane, the apical-basal polarity is perturbed or even reversed when GSK3 is inhibited, as revealed by P-cadherin and Klk7 immunostaining (Fig. 2g middle and right). These results indicate that GSK3 regulates ECM expression and basement membrane formation to modulate the epidermal cell apical-basal polarity. Accordingly, abrogation of apical-basal polarity by inhibition of GSK3 in newborn mouse skin explant cultures results in significantly decreased hair regeneration compared to the control (Fig. 2h).

Because Tgfb expression is also increased from 6h to D2 and colocalizes with Gsk3 and Collagens (Fig. 2a–c), we treated the D0 newborn mouse skin explant cultures with Tgfb recombinant protein which led to significantly increased mRNA expression of Gsk3a, Gsk3b, Col1a1, and Col4a1 at D2 (Supplementary Fig. 2j). While inhibition of Tgfb activity at D0 led to dramatically decreased mRNA expression of these genes at D2 (Fig. 2f and Supplementary Fig. 2j), suggesting that Tgfb regulates Gsk3 and ECM expression. Inhibition of Tgfb resulted in significantly lower Collagen I and IV expression in the specified dermal cells and impaired basement membrane formation compared to the controls (Fig. 2g and Supplementary Fig. 2k), smaller epidermal aggregates, and fewer dermal cell attachments (Supplementary Fig. 2i).

Inhibiting the Tgfb pathway also results in dramatically decreased b1-integrin expression in the outer epidermal aggregate layer at D2 and D3 in newborn mouse skin explant cultures (Supplementary Fig. 2m), suggesting a decreased EMI. Immunostaining showed that E-cadherin is not polarized in the outer layer of the Tgfb inhibition group compared to the control (Supplementary Fig. 2n). Intriguingly, P-cadherin has a higher expression in the inner layers but Klk7 has a higher expression in the outer layers of the epidermal aggregate in the Tgfb inhibition group (Fig. 2g and Supplementary Fig. 2n), indicating the apical-basal polarity has been inverted. This confirms that Tgfb regulates CMU formation (Fig. 2g schematic). Likewise, transplantation of Tgfb-inhibited cells to the back of nude mice results in significantly fewer regenerated hairs compared to the control (Fig. 2h). Together, this provides further compelling evidence that CMU formation in reconstituted newborn mouse skin explant morphogenesis influences the outcome of hair regeneration upon transplantation.

### PKR–PKC axis promotes apical-basal polarity formation in epidermal cells positioned in the outer layer of cell aggregates

**Fig. 3:**
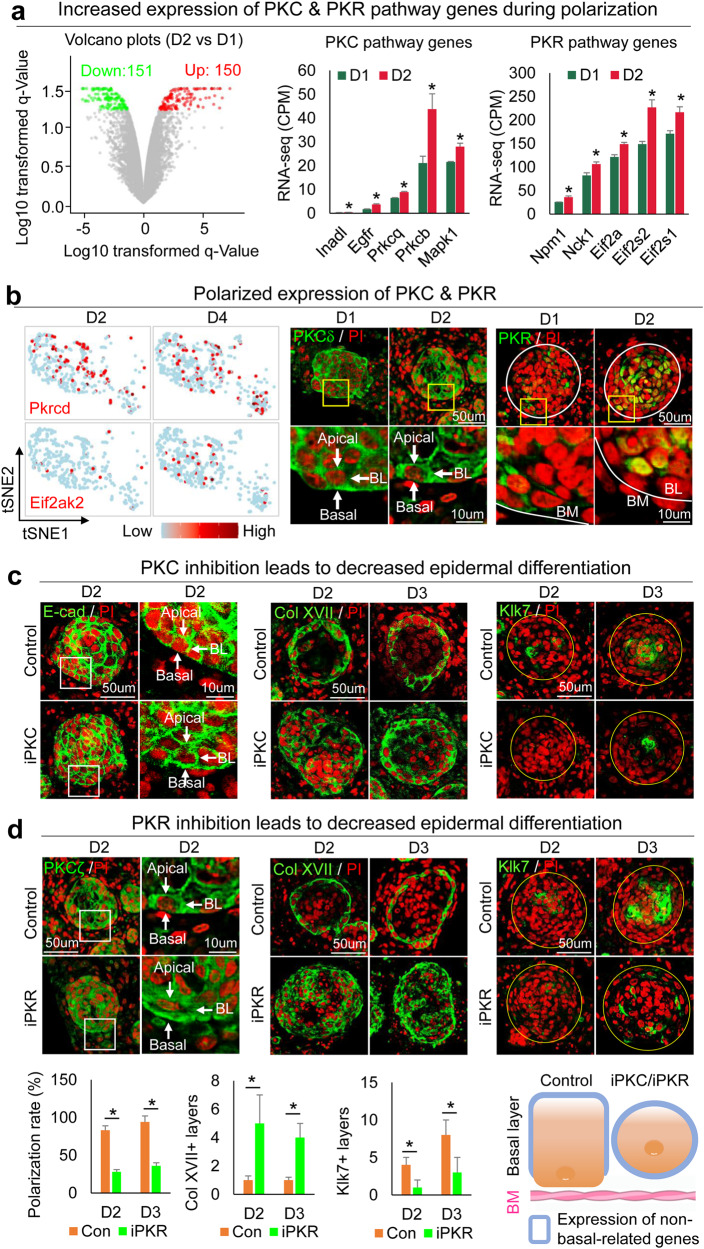
PKR-PKC promoting polarization of epidermal cells toward the formation of the epidermal cyst in newborn mouse cell cultures. **a** RNA-seq analysis shows PKC and PKR pathway genes upregulated from D1 to D2. *p < 0.05, *n* = 2. **b** TSNE plots and immunostaining of representative PKC (Pkrcd) and PKR (Eif2ak2) pathway gene expression in skin organoid cultures. **c** Immunostaining of E-cadherin, Collagen XVII, and Klk7 shows inhibition of PKC (iPKC) leads to decreased polarization at D2 and D3. **d** Immunostaining and quantification of PKCζ, Collagen XVII, and Klk7 show inhibition of PKC (iPKC) leads to decreased polarization at D2 and D3. *p < 0.05. *N* ≥ 3.

Since polarization occurs in the epidermal aggregate, we then studied how epidermal cell polarization influences CMU formation. Gene ontology analysis of D2 upregulated genes in newborn mouse skin explant cultures showed that highly enriched genes are involved in keratinization, keratinocyte differentiation, epidermal cell differentiation, etc. (Fig. 3a, Supplementary Fig. 3a-b, and Supplementary Table 3), which mainly include PKC and PKR pathway genes that may regulate epidermal differentiation (Fig. 3a and Supplementary Fig. 3c). Representative PKR and PKC pathway genes are expressed in both basal and suprabasal cells, and the number of expressing cells is increased from D2 to D4 in newborn mouse skin explant cultures as seen in tSNE plots (Fig. 3b and Supplementary Fig. 3d). Immunostaining confirms that PKCa and PKCδ are expressed in both basal and suprabasal cells. D2 epidermal aggregate basal cells show polarized expression (Fig. 3b and Supplementary Fig. 3d). PKR expression is increased from D1 to D2, particularly in the suprabasal cells (Fig. 3b).

To test if PKC regulates epidermal apical–basal polarity, we inhibited PKC function by adding a specific inhibitor, Bisindolylmaleimide I, to the culture at D1 newborn mouse skin explant cultures. K14 immunostaining shows that epidermal cell aggregation is not influenced (Supplementary Fig. 3f), but the polarized expression of E-cadherin in the basal layer is abolished by this treatment at D2 and D3 (Fig. 3c and Supplementary Fig. 3f). Collagen XVII and P-cadherin are increased but Klk7 is decreased in the epidermal cyst in the PKC inhibition group compared to that of the control group at D2 and D3 (Fig. 3c and Supplementary Fig. 3f). To examine if PKR regulates PKC at the mRNA level, we inhibited PKR function with imidazolo-oxindole and found that Prkca expression did not change significantly (Supplementary Fig. 3e). However, inhibiting PKR blocked PKC polarization (Fig. 3d left), indicating that PKR regulates PKC at the protein level. Furthermore, inhibiting PKR led to increased Collagen XVII and decreased Klk7 expression (Fig. 3d), and abolished polarization of E-cadherin and P-cadherin in the basal layer of the epidermal aggregate (Fig. 3d and Supplementary Fig. 3f). These findings indicate that PKR regulates PKC polarization which contributes to apical-basal polarity (Fig. 3d and Supplementary Fig. 3g). To confirm this, we added both PKR and PKC inhibitors to the D0 culture and observed decreased epidermal differentiation, without obvious influence of cell apoptosis at D2 and D3 newborn mouse skin explant cultures (Supplementary Fig. 3h-i).

### IFNr suppresses PKR–PKC axis in the epidermal basal cells to maintain their multipotency

**Fig. 4:**
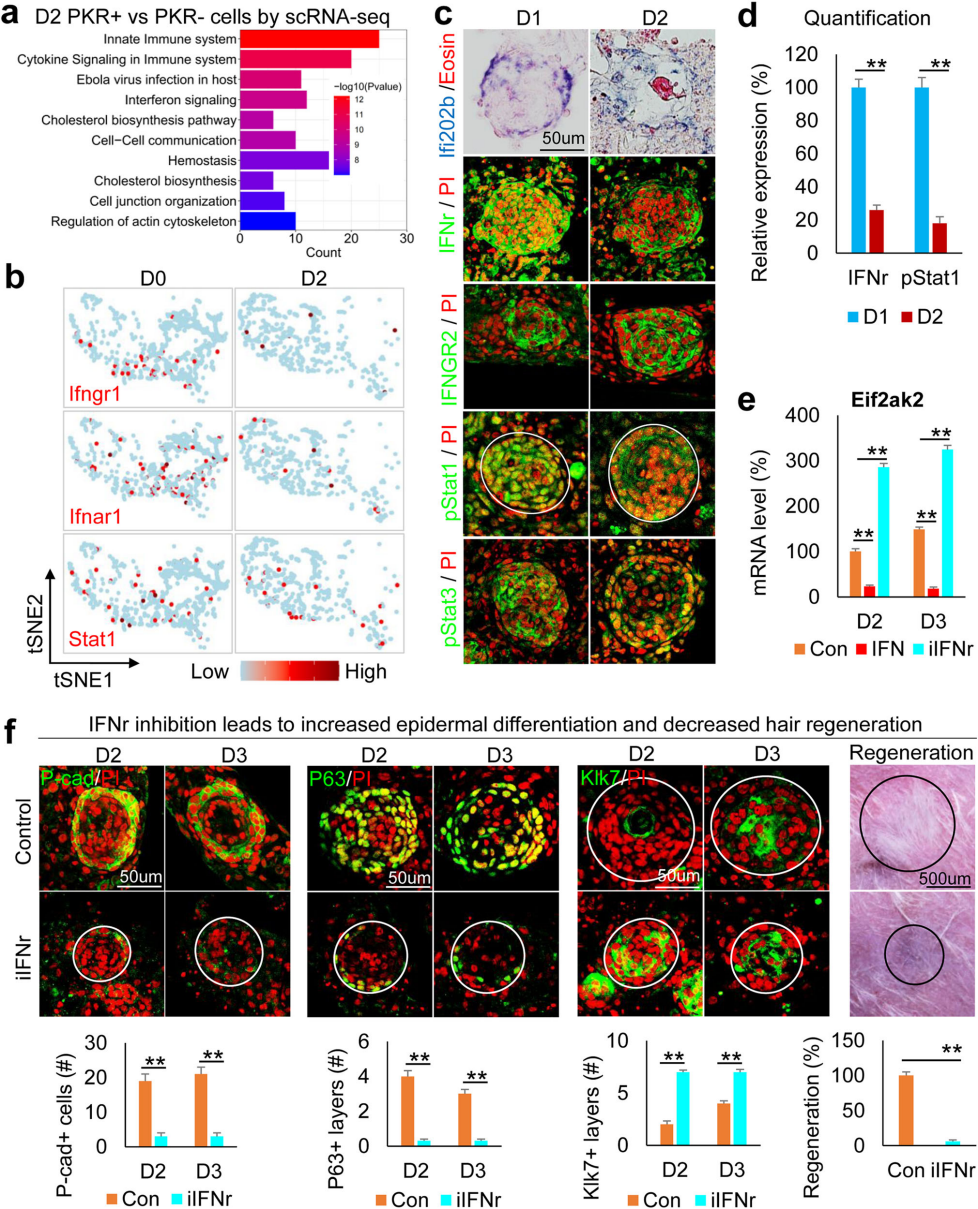
IFNr negatively regulates PKR–PKC in the newborn mouse epidermal aggregates. **a** Ontology of gene expression in PKR+ vs. PKR− cells in D2 culture. **b** TSNE plots show representative interferon pathway genes in epidermal cells. **c** Immunostaining and in situ hybridization for representative interferon pathway gene expression in D1 and D2 cultures. **d** Quantification of pStat1 expression. **p < 0.01. N ≥ 3. **e** Eif2ak2 expression after addition or inhibition of IFNr. **p < 0.01. *N* = 3. **f** Immunostaining and quantification show that inhibition of IFNr results in decreased P-cadherin and P63 but increased Klk7 expression at D2 and D3. Hair regeneration is significantly decreased after transplantation of iIFN-treated cells compared to the control. **p < 0.01. *N* = 5.

We next sought to identify upstream regulators of the PKR–PKC axis that control apical-basal polarity formation. We compared gene expression in PKR+ vs. PKR–epidermal cells by analyzing the D2 (polarized stage) scRNA-seq data of newborn mouse skin explant cultures. The top four GO terms are all related to interferon pathways in PKR+ vs. PKR-cells (Fig. 4a and Supplementary Table 4). Surprisingly, tSNE plots revealed that the number of cells expressing key interferon pathway genes (e.g., Ifngr1, Ifnar1, and Stat1) decreased from D0 to D2 (Fig. 4b). Immunostaining and in situ hybridization showed that Ifi202b, IFNr, IFNGR2, pSTAT1, and pSTAT3 are decreased from D1 to D2 newborn mouse skin explant cultures, implying that the interferon pathway may negatively regulate the polarization process (Fig. 4c, d). Adding IFNr recombinant protein suppresses Eif2ak2 (encodes PKR) whereas inhibiting IFNr enhances Eif2ak2 expression (Fig. 4e). These results suggest IFNr negatively regulates Eif2ak2 expression. Inhibiting IFNr led to dramatically smaller aggregate formation compared to the control (Supplementary Fig. 4a). The expression of P-cadherin, P63, Collagen XVII, and E-cadherin is significantly decreased and the expression of Klk7 is significantly increased in the IFNr-treated group compared to the control group (Fig. 4f and Supplementary Fig. 4b, c), indicating that the IFNr is required to maintain the multipotency of the CMU. Perturbed CMU formation in the IFNr-treated cultures led to significantly decreased hair regeneration upon transplantation compared to the control (Fig. 4f).

Interestingly, we also observed that inhibiting IFNr led to disrupted basement membrane (Laminin+) formation (Supplementary Fig. 4d) and decreased Collagen I assembly surrounding the epidermal aggregate (Supplementary Fig. 4e), implying that epidermal cells can interact with the specified dermal cells. In addition, without the expression of ECM in the specified dermal cells in the IFNr-treated group, the ECM receptor b1-integrin is dramatically decreased in the basal layer of the epidermal aggregate (Supplementary Fig. 4f). These results further confirm the epidermal-dermal communications suggested by CellChat analyses.

### VEGF mediates dermal cell attachment to the epidermal cyst

**Fig. 5:**
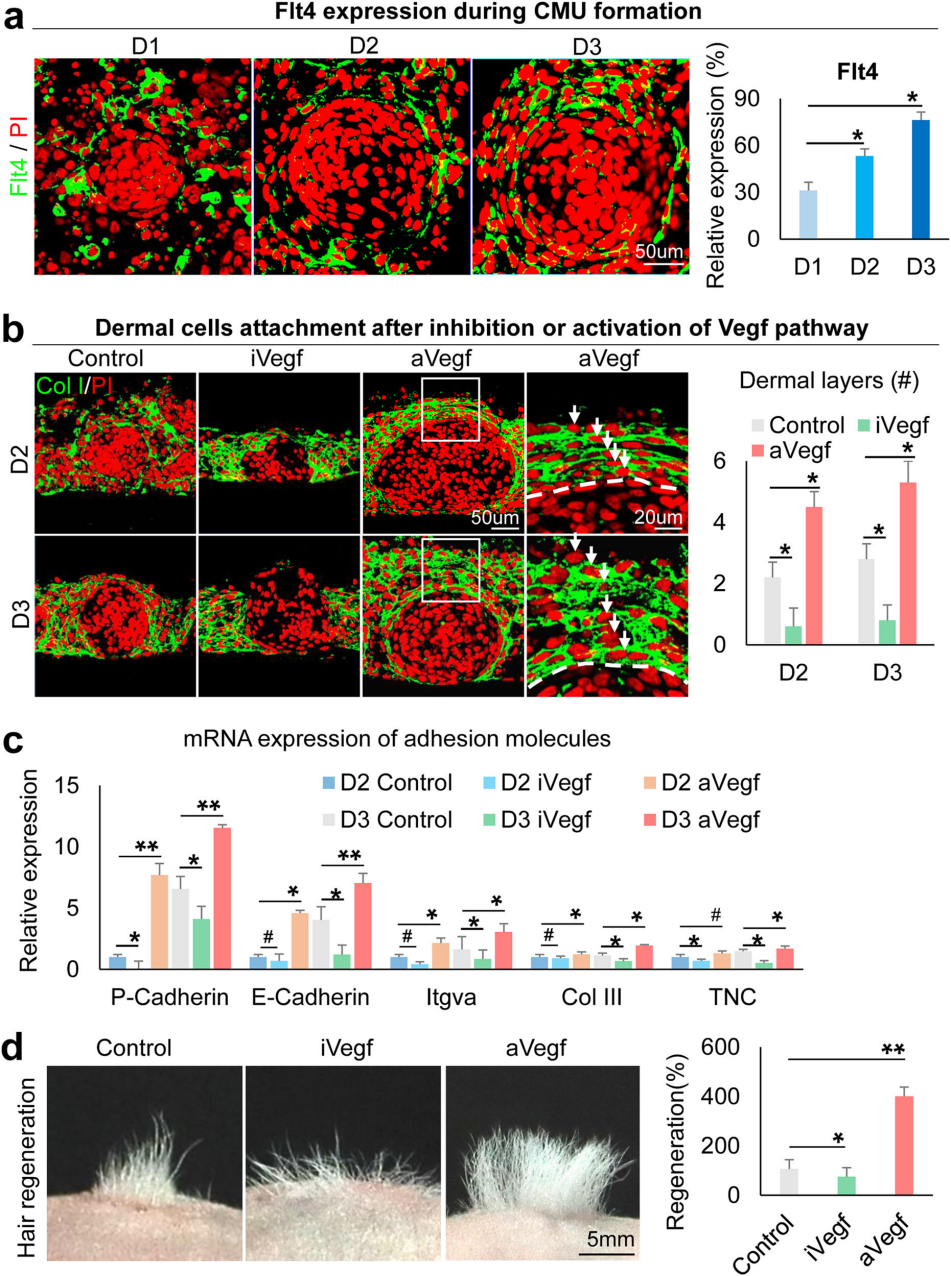
VEGF regulates dermal cell attachment during CMU formation in newborn mouse cell cultures. **a** Immunostaining shows Flt4 expression is gradually increased in dermal cells surrounding the epidermal aggregate at D1–D3. *p < 0.05. *N* ≥ 3. **b** Immunostaining for Collagen I shows that inhibition of VEGF results in decreased dermal cell attachment and vice versa at D2 and D3. *p < 0.05. *N* ≥ 3. **c** qRT-PCR shows cell adhesion expression after VEGF inhibition or activation. *p < 0.05. #, no statistical significance. *N* = 3. **d** Hair regeneration with transplantation of iVegf or aVegf-treated cells. I, inhibition; a, activation. *p < 0.05 and **p < 0.01. *N* = 4.

Our previous study confirmed that dermal cells are required for epidermal cell self-organization^14^. RNA-seq analysis showed VEGF signaling pathway genes are significantly increased at D2 vs. 6h newborn mouse skin explant cultures (Supplementary Fig. 5a). We examined the expression of Flt4, which is significantly upregulated among VEGF signaling pathway receptors (Supplementary Fig. 5a). Immunostaining shows that Flt4 is gradually increased in the specified dermal cells from D1 to D3 in newborn mouse skin explant cultures (Fig. 5a). We then treated the cultures with Axitinib (an inhibitor of VEGF receptors) or VEGF recombinant protein to evaluate the function of VEGF signaling. Intriguingly, we observed that the addition of VEGF recombinant protein results in significantly more dermal cell layers attached to the epidermal aggregate compared to the control, and vice versa in the VEGF inhibition groups. Immunostaining for K14, Collagen IV, and Laminin shows that VEGF inhibition leads to the formation of a smaller aggregate and an abolished basement membrane compared to the control (Fig. 5b and Supplementary Fig. 5b, c). qRT-PCR reveals that adhesion molecules expressed in both epidermal cells (e.g., P-cadherin, E-cadherin, and Itgva) and dermal cells (e.g., Collagen III and Tnc) are significantly increased in D2 and D3 after addition of VEGF recombinant protein (Fig. 5c). These data indicate that VEGF signaling regulates dermal cell attachment during CMU formation (Supplementary Fig. 5d). We also validated that exogenous inhibition or addition of VEGF leads to decreased or increased Flt4 expression in specified dermal cells, respectively (Supplementary Fig. 5e). Transplantation of VEGF recombinant protein-treated cultures into nude mice results in significantly more hair formation compared to the control, and vice versa in the VEGF inhibition group (Fig. 5d). This further suggests that proper dermal cell attachment is required for hair regeneration.

### Restoring the hair regeneration ability of adult mouse cells by re-establishing morphogenetic competence

**Fig. 6:**
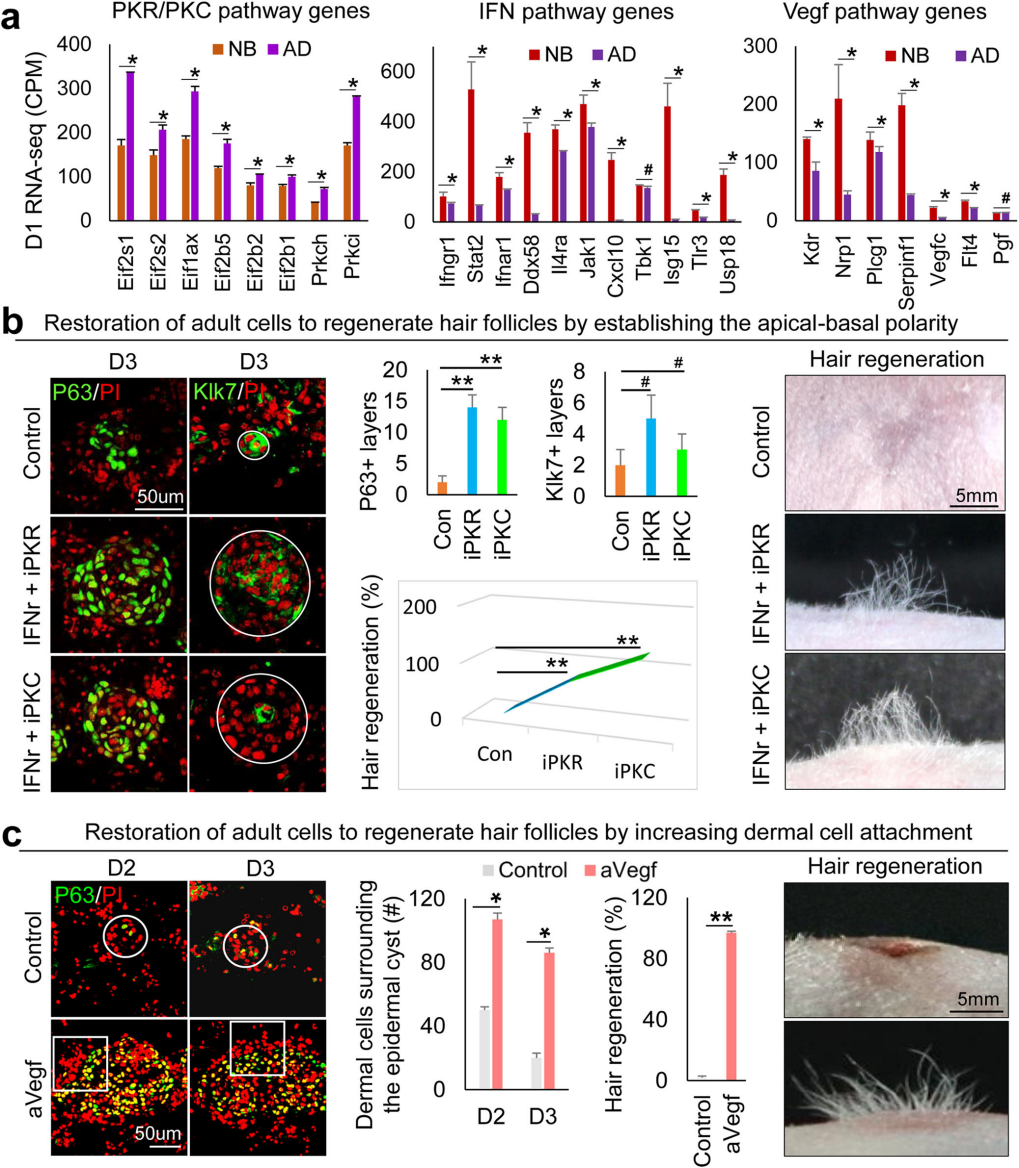
Restoration of the adult mouse cells to form CMU after environmental reprogramming. **a** RNA-seq comparison of PKR, PKC, IFN, and VEGF pathway genes between newborn and adult at D2. *p < 0.05. #, no statistical significance. *N* = 2. **b** Immunostaining and quantification of P63 and Klk7 expression show that inhibition of PKR or PKC results in decreased epidermal cell differentiation in adult mouse cell cultures at D2 and D3. Increased hair regeneration with transplantation of IFNr+iPKR- or IFNr+iPKC-treated adult mouse skin cells. **p < 0.01. #, no statistical significance. *N* = 8. **c** Increased dermal cell attachment and hair regeneration after the addition of VEGF recombinant protein (aVEGF) compared to the controls. *p < 0.05, **p < 0.01, # no significant difference. *N* = 4.

Adult mouse cells lose hair regenerative abilities after culture^13,14^. This may be due to excessive epidermal differentiation and weak EMI that leads to failed CMU formation. Using RNA-seq analysis, we observed that PKC and PKR pathway genes are significantly increased, but IFN and VEGF pathway genes are significantly decreased in D1 adults compared to newborn (unpolarized stage) mouse cell cultures (Fig. 6a). Immunostaining confirmed the increased expression of PKC and PKR, and decreased expression of IFNr and Flt4 in adult mouse cells compared to newborn mouse cells (Supplementary Fig. 6a). Using a modified environmental reprogramming protocol (Supplementary Fig. 6b), we next tried to reactivate the adult mouse cells to form CMU by modulating these molecules. We observed that PKR or PKC inhibition results in larger aggregate formation without the addition of growth factors in adult mouse cell cultures. However, these conditions do not achieve the levels seen in newborn mouse cell cultures (Supplementary Fig. 6c). With IFNr addition and PKR/PKC inhibition, the skin cysts show a polarized structure including basal layers (P63+ and Collagen XVII+) and suprabasal layers (Klk7+), compared to the control groups without apical–basal polarity formation (Fig. 6b and Supplementary Fig. 6d). The enlarged skin cyst in IFNr + iPKR (or iPKC) cultures is not due to cell proliferation (Ki67+) but due to increased cell adhesion (E-cadherin + ) (Supplementary Fig. 6d). A more complete basement membrane (Laminin+) is formed in the restored group compared to the control (Supplementary Fig. 6d). Transplantation of the restored adult mouse skin explant cultures to nude mice results in significantly more hair regeneration compared to the control (Fig. 6b). Using a similar strategy, we successfully restored basement membrane formation and dermal cell attachment by adding VEGF recombinant protein to the adult cell culture (Fig. 6c and Supplementary Fig. 6e, f). This results in significantly more hair regeneration compared to the control (Fig. 6c). These data suggest that the reestablishment of CMU in the mouse skin explant culture is sufficient to restore the regenerative ability of adult cells to form hair follicles upon transplantation.

**Fig. 7:**
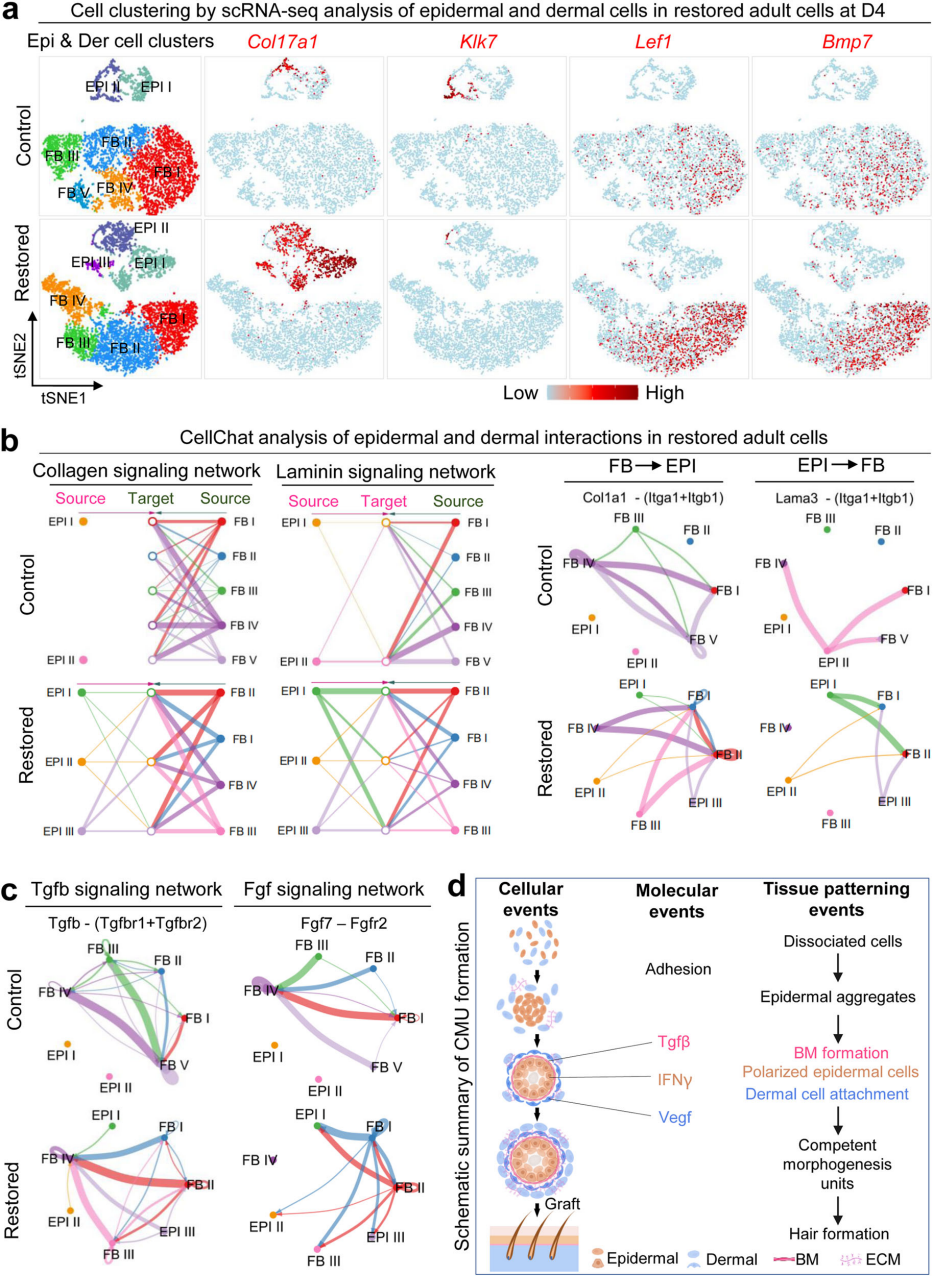
ScRNA-seq analysis of epidermal–dermal cell communication in restored adult cell cultures after environmental reprogramming. **a** ScRNA-seq analysis shows an increased number of basal cells (Col17a1+), DP cells (Lef1+, Bmp7+), and a decreased number of suprabasal cells (Klk7+) in restored adult cells. **b** ECM mode of CellChat analysis shows increased EMI in restored adult cells. False discovery rate <0.05 and Log2 fold change >1. **c** Secreted mode of CellChat analysis shows enhanced Tgfb and Fgf signaling network between epidermal and dermal cells in the restored group. **d** Summary diagram shows the mechanism of CMU formation.

In addition to examining the morphological characteristics of CMU formation, we also checked the molecular identities of the restored adult mouse explant cultures by performing scRNA-seq analysis. Since CMU formation mainly involves EMI, we isolated adult mouse epidermal and dermal cells which were distributed into different sub-clusters (Fig. 7a, Supplementary Fig. 7a, and Supplementary Table 5). TSNE plots reveal that the restored adult mouse skin explants have more basal cells (Col17a1+) and fewer suprabasal cells (Klk7+) than the control group, implying successful maintenance of epidermal multipotency. The number of Lef1+ and Bmp7+ dermal cells (which may constitute the prospective dermal condensate) is also increased in the restored adult mouse skin explants (Fig. 7a). CellChat analysis reveals an increased number of interactions and enhanced weights/strength in restored adult mouse skin explants compared to the control (Supplementary Fig. 7b and Supplementary Table 6), indicating intensive EMIs after environmental reprogramming. More Collagen and Laminin signaling networks are formed between epidermal and dermal cells in the restored adult mouse skin explants (Fig. 7b). For example, dermal cell-secreted Col1a1 signaling can be sensed by Itga1 and Itgb1 expressed by the epidermal cells in the restored group but not in the control group. The epidermal cell-expressed Lama3 can be received by FB I and FB II in the restored group but less in the control group.

CellChat secreted mode analysis reveals stronger interactions of ligands and receptors between epidermal and dermal cells in restored adult mouse skin cells compared to the control (Supplementary Fig. 7c). For example, the Tgfb signaling secreted by dermal fibroblasts can be sensed by Tgfbr1 and Tgfbr2 expressed in the epidermal cells in the restored group but not in the control group (Fig. 7c and Supplementary Fig. 7d). FGF signaling which is required for hair regeneration can be secreted by dermal cells and received by epidermal cells in the restored group but not in the control group (Fig. 7c). These data suggest that the EMI is dramatically enhanced at the molecular level after environmental reprogramming. Indeed, scRNA-seq analysis and immunostaining showed an increased number of cells that express dermal papilla markers including Wif1a, Lef1, Sox2, and Rspo3 in the restored group compared to the control (Supplementary Fig. 7e, f). The restored adult mouse skin explants more closely resemble that of the newborn mouse cell cultures, facilitating CMU formation and hair regeneration upon transplantation.

### Enhancing the hair regeneration ability of human cells with what we learned from the mouse cells

**Fig. 8:**
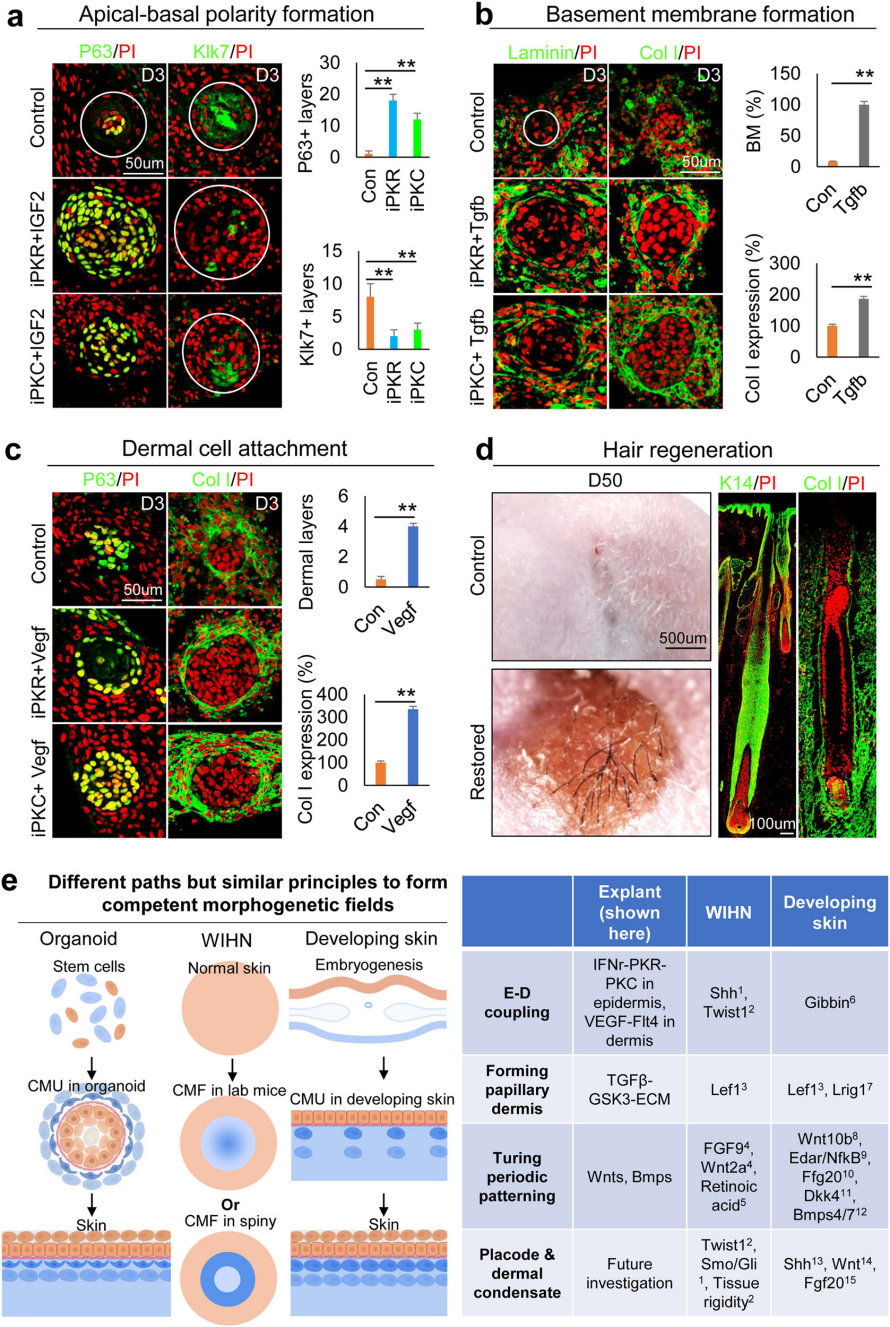
Restoration of the human fetal scalp cells to form CMU after environmental reprogramming. Epidermal and dermal cells were harvested from the 24-week human fetal scalp with the same culture condition as the mouse skin explant culture. **a** Immunostaining and quantification of P63 and Klk7 expression show that inhibition of PKR or PKC results in decreased epidermal cell differentiation in human fetal scalp cell cultures at D3 compared to the controls. **p < 0.01. *N* = 8. **b** Immunostaining and quantification of Laminin and Collagen I expression show that addition of Tgfb led to a more complete basement membrane formation compared to the controls. **p < 0.01. *N* = 8. **c** Increased dermal cell attachment after the addition of VEGF recombinant protein compared to the controls. **p < 0.01. *N* = 8. **d** Hair regeneration after environmental reprogramming. Immunostaining for K14 and Collagen I which only stain human cells in the reconstituted skin. *N* = 4. **e** A comparing skin explant, wound-induced hair regeneration (WIHN), and normal developing skin shows that all of them have to form the competent morphogenetic field (CMF) before Turing patterning can take place. They may show different molecular paths but similar principles were used. See references in supplementary information.

To further test if the principles of CMU formation learned from newborn mouse cell self-organization can be applied to restore human cells’ ability to regenerate hair follicles from skin explants, we modified the culture conditions (Supplementary Fig. 6b) to induce CMU formation from human fetal scalp cells. P63 and Klk7 immunostaining showed that the apical-basal polarity can be established by adding PKR or PKC inhibitors to the cultures of 24-week fetal scalp cells (Fig. 8a). These human epidermal aggregates have enlarged size, stronger cell adhesion, and increased stemness, as indicated by representative markers (Supplementary Fig. 8). Moreover, the addition of TGFβ recombinant protein induce basement membrane formation and increased Collagen I expression (Fig. 8b). Addition of VEGF significantly increased dermal cell attachment surrounding the epidermal cyst compared to the control group (Fig. 8c). Transplantation of these human skin explants to the back of nude mice resulted in prominent hair regeneration, compared to the control which does not have visible hairs (Fig. 8d). Antibodies against human K14 and Collagen I confirmed the neogenic hair follicles are derived from donor cells (Fig. 8d). These data further suggest that the formation of similar epidermal-dermal spheroids in human skin explant culture can enhance hair regeneration upon transplantation onto the nude mice back skin.

## Discussion

In this work, we revealed that dissociated skin progenitor cells from newborn mouse skin can self-organize to generate epithelial cysts surrounded by 3–4 layers of dermal cells. We named these “epidermal-dermal spheroids” and demonstrated they represent a new multi-cellular state that is required for further morphogenesis to establish tissue patterns. Based on these functions, we name these spheroids “competent morphogenetic units (CMU)”. Using scRNA-seq analyses, we report the emergence of cell clusters and molecular signaling modules during this self-organizing process. This transcriptome is compared with adult mouse cell skin explants which fail to undergo morphogenesis. Thereby we used these molecular differences to rescue hair morphogenesis in adult mouse skin explant cultures. Furthermore, we apply what we learned from mouse cells to human fetal scalp cells and show they can form hairs after transplanting to the nude mice. Finally, we distilled the requirements to establish morphogenetic competence by comparing the different paths to form tissue patterns in these cultured explants, developing embryonic skin, and WIHN (Fig. 8e).

In developing mouse skin, signals from the epidermis and dermis communicate, using back-and-forth feedback signals to coordinate their behavior and future gene expression. This signaling activity establishes the epidermal placodes and dermal condensations for hair formation^22,23^. In the wound-induced hair neogenesis of laboratory mice^1^, hairs form in the center of the wound field, because the condition for de novo patterning rises above the threshold. The skin of spiny mice has lower tissue rigidity which causes the topology of the morphogenetic competent zone to start from the periphery of the wound bed which propagates toward the center of the wound bed^24^. Thus, the basic requirement is to meet the threshold for Turing periodic patterning, and the topology of the morphogenetic field can vary depending on the cellular context found in culture, developing skin, or wound beds.

One of the fundamental shared principles is the establishment of EMI, allowing a series of programmed, sequential, and reciprocal (complex and multiphase) communications between the epidermal and dermal cells to take place. Since the cultures started from dissociated epidermal and dermal cells, these EMIs have to be re-established. This process is demonstrated by scRNA and CellChat analyses: an increase of EMI from D2 to D4 in newborn explants (Fig. 1), scanty EMI in adult explants, and the establishment of EMI in restored adult and human explants (Figs. 6–8). It is interesting to note the cellular interactions and molecular pathways in the formation of EMI are different from those used in embryonic development. Thus we need to learn more about how these “morphogenesis in vitro” processes take place.

In this reconstituted skin explant culture using newborn mouse cells, dissociated epidermal cells adhere to form aggregates among dissociated dermal cells that remain dispersed between these aggregates^25^. Epidermal cells show equal expression of stem cell markers (e.g., P-cadherin+ and Collagen XVII+). Later, cells in the outer aggregate maintain their stem cell identity during culture at D2–D3, while cells in the inner aggregate begin to differentiate into suprabasal layers (e.g., Fabp5+ and Klk7+). This process is illustrated in the epidermal clustering by scRNA-seq from D2 to D4 when a basal cell cluster and three suprabasal cell clusters form.

This process resembles the developmental process of epidermal differentiation which is regulated by PKC activity^26,27^. What signaling modulates PKC activity in these explants? Our study revealed that PKR positively regulates PKC activity at the protein level. Loss of PKR activity is associated with inhibition of PKC polarization, suggesting both PKR and PKC promote epidermal cell polarization. In addition, PKR is known to promote cell apoptosis^28,29^. Interestingly, while the best-described transcriptional motif in the PKR promoter is an IFN-stimulated response element (ISRE) and PKR can be activated by pro-inflammatory cytokines (e.g., IFNr)^30^, we identified that epidermal cells secrete IFNr to negatively regulate PKR expression in CMU. Epidermal keratinocytes have been shown to produce interferons^31^. Unexpectedly, we found IFNr is expressed and used in these explants to maintain epidermal cell multipotency, a pathway not used in developing skin.

Without dermal cells, epidermal cells only form small aggregates, implying that communication with dermal cells is essential for epidermal cell polarization. What dermal signals are used in these explants? We probed the upstream regulators of ECM production by comparing Col4a1+ and Col4a1− cells and found that TGFb signaling genes are highly expressed in Col4a1+ cells at the polarization stage at D2. This cell population mainly corresponds to FBs II. TGFb is known to induce ECM expression in fibroblasts^32^. Indeed, we observed that Tgfb receptors (1 and 2) and Smads (2 and 3) are highly expressed in the specified dermal cells, suggesting that the Tgfb pathway is activated during CMU formation. We also found that Gsk3 is enriched in Tgfb+ FB II. Inhibition of GSK3 leads to the activation of Wnt signaling^33^, which is known to activate MMPs^34^. Blocking the GSK3 function here perturbs CMU formation by disrupting ECM assembly, causing an inverted apical-basal polarity phenotype, similar to that seen with Tgfb inhibition (Fig. 2g). It has also been reported that GSK3 accounts for TGF-β-inducible Ser204 phosphorylation^35^, and regulates ECM remodeling in chondrocytes^36^. Thus TGFb-GSK3-ECM signaling from the FB II produces ECM for basement membrane formation required for CMU formation. We also observed the continuous addition of dermal cells to the cyst shell from 1 to 4 cell layers in a dermal condensation-like process in developing skin^22,23^. Here VEGF appears to play a major role in this dermal cell recruitment process.

Other than the skin, there are cultures cultured under certain cytokine and matrix conditions. Some of these explant or organoid cultures have been observed to exhibit transient embryoid body-like structures with a cystic (or lumen, tubule) configuration before they further develop into mini-organs^37–40^, whether the epithelia are derived from ectoderm, endoderm, or mesoderm. Scientists tend to name this process as self-organization. Using the current study as an example, we can appreciate morphogenesis in vitro have different paths. With the formation of epidermal-dermal coupled spheroids as one of the morphological markers, we can modify in vitro culture conditions to enhance the regenerative ability of the explant or organoid cultures in vivo. As we appreciate there are multiple paths to establish tissue patterns^4^ (Fig. 8e), we need to study more to establish the principles of “in vitro morphogenesis” in the future. For basic science, they will help reveal mechanisms of self-organization. For the application side, they will help identify more potential therapeutic targets.

## Methods

### Mice

K14H2BGFP mice were a kind gift from Dr. Elaine Fuchs’s laboratory at Rockefeller University. CD1 and nude mice were purchased from GemPharmatech (Jiangsu, China). Mice were kept at 26 °C with a 12-h light cycle, with food and water ad libitum. All performed procedures were approved by the Institutional Animal Care and Use Committees of Chongqing University. Euthanasia was performed on the mice using the cervical dislocation when we harvested the skin samples. To minimize potential discomfort and pain, each mouse was properly anesthetized with Ketamine (50–100 mg/kg, IM) and Xylazine (5–10 mg/kg, IM) prior to the procedure.

### Bulk RNA-seq analysis

The raw data of D0, 6h, D1, D2, and D4 samples were downloaded from the GEO database (accession No. GSE86955) which we generated. SRA Toolkit and Fastp were used for preprocessing the raw data, then RNA sequence alignment and assembly were carried out by HISAT2 and StringTie. The differentially expressed genes (DEGs) were identified by the R package DESeq2 v1.32. False discovery rate <0.05 and Log2 fold change >1 were set as the threshold to identify DEGs. ClusterProfiler v4 was used for analyzing the gene set enrichment (GO and KEGG).

### ScRNA-seq analysis

The D0, D2, and D4 newborn mice skin explant cultures were collected for single-cell RNA sequencing. Seurat v 4.1.2 was used for quality control, dimensionality reduction, and clustering. For each sample dataset, we filtered the expression matrix by the following criteria: (1) cells with gene count less than 500 or with top 2% gene count were excluded; (2) cells with top 2% unique molecular identifiers (UMIs) count were excluded; (3) cells with mitochondrial content >5% were excluded; (4) genes expressed in less than 5 cells were excluded. The top 20 principal components were selected for tSNE and UMAP reduction. The marker genes of each cluster were identified by the seurat functions FindNeighbours and FindClusters. The single R package and Cell Marker online database were used for the definition of each cell cluster. Visualization of genes was performed by seurat v4 and ggplot2 v3. D0 or D4 epidermal cell clusters (suprabasal cells and basal cells) or dermal cells were selected for pseudotime analysis. The R package monocle v3 was used for establishing the lineage trajectory of different cell clusters. After the annotation of cell clusters, four groups of dermal cells (FBI–FB IV) and two groups of epidermal cells (BC, SBC) were selected for cell communication analysis. Representative signature genes were used to annotate epidermal and dermal subclusters (Supplementary Fig. 1c). CellChat R package v1.1.3 was used to analyze the ligand-receptor interaction. “ECM-receptor” and “Secreted signaling” modes were selected for the epidermal–dermal interaction analysis.

### Primary cell culture

Newborn mouse cell skin explant culture. The method for explant culture using primary cells can be referred to our previous publication^16^. Briefly, cells were derived from the dorsal skin of neonatal mice within 24 h of birth or 6-month-old female CD1 mice. The mouse skin was floated in 0.25% trypsin solution at 4 °C to separate into the dermis and epidermis. The epidermis was cut with scissors, pipetted, and filtered through a 70 µm cell strainer followed by wash and centrifugation. The dermis was digested in 0.35% collagenase for 20 min, then filtered through a 70 µm cell strainer followed by wash and centrifugation. The dissociated epidermal cells and dermal cells were re-mixed at a ratio of 1:9 and were dropped onto the upper chamber of a transwell culture insert at a high density, and the lower chamber was filled with 700ul DMEM/F12 (Corning) culture medium containing 10% FBS (Gibco). The cells were cultured in a humidified atmosphere containing 5% CO_2_ at 37 °C, with the culture medium being changed every other day.

The above method can be used for progenitors derived from different sources. Adult mouse cell skin explant culture. The growth factors were added from D0 to D4, followed by adding MMP14 recombinant protein from D4 to D7. PKC inhibitors were added throughout the cultivation period. The cells were cultured in a 5% CO_2_ at 37 °C incubator, with the culture medium being changed every other day.

### Human skin cell isolation

We performed human studies using cells from 24-week abortive fetal scalp samples which have a better phenotype resembling the newborn mouse cell explant culture. The dermal cells in the human skin explant culture system are derived from a 24-week-old abortive fetal scalp, and the epidermal cells are derived from the foreskin tissue of healthy children. We have complied with all relevant ethical regulations including the Declaration of Helsinki when using human samples. All human specimen usage and performed procedures were approved by the Institutional Review Board of Chongqing University. All written informed consent was obtained from the guardians. Each experiment was repeated four times and they all were successful, with some differences in hair regeneration after transplantation.

The fetal scalp was floated in 0.25% trypsin solution at 4 °C to separate into the dermis and epidermis. The dermis was digested into single cells in 0.35% collagenase (#LS004197, Worthington, USA) solution at 37 °C water bath for 90 min. The red blood cells were removed using RBC lysis buffer (#RT122-02, Tiangen, Beijing).

The epidermis was separated from the adult’s foreskin which was digested in 0.25% dispase II (#D6430, Solarbio, Beijing) solution at 4 °C overnight. The separated epidermis was cut with scissors, pipetted, and filtered through a 70 µm cell strainer. The dissociated epidermal cells and dermal cells were mixed at a ratio of 1:5 and were dropped onto the upper chamber of a transwell culture insert, and the lower chamber was filled with 700 µl DMEM/F12 (Corning) culture medium containing 10% FBS (Gibco). The cells were cultured in a 5% CO_2_ at 37 °C incubator, with the culture medium being changed every other day.

### Mouse cell isolation for scRNA-seq

To dissociate the skin explants into single cells, the cell cultures (*n* = 6 per sample) were incubated with TrypLE for 30 min at 37 °C and gently shaken at 60 rpm on a shaker, and DNase I was added to further eliminate the aggregates formed during lysis. Cell debris was filtered out using a 40 µm cell strainer, the cells were identified as live or dead by Taipan Blue staining to ensure that over 95% of the cells were viable. All samples were processed in accordance with the provisions of 10× Genomics ChromiumTM Single Cell 30 Reagent Guidelines v3 Chemistry. The quality control and cDNA quantification were performed using Agilent High Sensitivity DNA Kit. The cells were sequenced using Illumina NovaSeq6000 with a depth of >20,000 reads per cell.

### ScRNA-seq annotation

We defined the basal cells (BC) based on the expression of K14 and Col17a1. Then, we defined three groups of suprabasal cells: SBC I (Fabp5, Krtdap, Krt6a), SBC II (Krt79, Lurap1l), and SBC III (Klk6, Klk7, Sprr1a) based on the expression of different keratins and other genes. In addition, we defined dermal cell clusters based on the expression of Vim and Col1a1, which are the core fibroblast genes. Due to the high heterogeneity of dermal fibroblasts (FB), we further refined their definition using different marker genes. FB I (Tnc, Thbs2, Dcn) and FB II (Col6a1, Lamc3) may represent dermal fibroblasts that are closer to the epidermis. In particular, through pseudo-time analysis of FB II (Supplementary Fig. 1G), we discovered that a subset of cells in FB II exhibit DP properties (Bmp4, Bmp7, Lef1, Ltbp1). FBIII specifically expresses Cspg4, which is a marker for differentiated fibroblasts217. FB IV specifically expresses Procr318, Manf419, and other inflammation-mediated proliferative fibroblast marker genes.

### H&E staining

Cell cultures were fixed in 4% PFA buffer at 4 °C overnight. Paraffin-embedded tissue was cut into 6 µm sections. For H&E staining, the samples were dewaxed and rehydrated, stained with hematoxylin and eosin for 2 min, then soaked in tap water for 5 min, and finally dehydrated, transparent, and sealed.

### Immunostaining

After dewaxing and rehydrating, the samples were antigen retrieved with pH 6.0 solution with citric acid and sodium citrate and blocked with 2% BSA at 37 °C for 1 h. The samples were incubated with primary antibodies at 4 °C overnight and then incubated with secondary antibodies at 37 °C for 2 h. DAPI (4’,6-diamidino-2-phenylindole) or PI (propidium iodide) was used to stain the nuclei. The primary and secondary antibodies used in the present study are listed in Supplemental Table 7.

### In situ hybridization

The method for in situ hybridization can be referred to our previous publications 1. Briefly, the paraffin sections were dewaxed, rehydrated with graded ethanol series, treated with proteinase K, acetic anhydride/triethanolamine, and then dehydrated with graded ethanol series. Then the sections were incubated with hybridization solution-diluted probes at 65 °C overnight. After being washed, the sections were incubated with Anti-Dig-AP conjugated antibody at 37 °C for 2 h, developed with NBT/BCIP (Roche) buffer overnight. The probes used in the present study are listed in Supplemental Table S8.

### RT-PCR

Total RNA was isolated with Trizol (TianGen) followed by extraction using an RNA simple Total RNA Extraction Kit (TianGen, DP419). To obtain cDNA, equal amounts of RNA were added to the reverse-transcriptase reaction mix (Takara, RR037A). Real-time PCR was performed in a total volume of 10 µl using the iTap™ Universal SYBR® Green Supermix kit (Bio-Rad) on a CFX96™ Real-Time PCR System (Bio-Rad). Primer sequences for target genes are listed in Supplemental Table 8, with Gapdh as an internal standard. Relative transcript levels of target genes were calculated by using Origin 8.0 software.

### Transplantation

Nude mice were prepared and draped with betadine solution under anesthesia. The skin was excised by 1 cm in diameter on both sides of the dorsal back. The cell cultures were transplanted onto the wound bed of nude mice. Three weeks after transplantation, the number of regenerated hairs was counted.

### Small molecule perturbation and recombinant protein treatment

A small molecule or recombinant protein was added to the culture medium 1 or 2 days before sampling unless specifically stated. The names, companies, catalog numbers, and concentrations of small molecules and recombinant proteins are listed in Supplemental Table 9. The aggregate size was calculated by using Imaris software or manually. The polarization rate and dermal cell attachment were counted manually. The basement membrane integrity and relative immunofluorescence intensity were calculated by using Image J.

### TUNEL assay

TUNEL staining was done by a Meilun One Step TUNEL Apoptosis Assay Kit (TRITC, #MA0224, Meilunbio, China) according to the manufacturer’s instructions. After being dewaxed and rehydrated, the samples were permeabilized with 20 µg/ml Proteinase K at 37 °C for 30 min, incubated with TUNEL detection solution (TdT Enzyme, 10×: TRITC-dUTP Labeling Mix = 1:9) at 37 °C in the dark for 60 min. Images were obtained by acquiring emission at 552 nm under a confocal microscope (Leica, Germany).

### Statistical analyses

Statistical analysis and data visualization were performed using Microsoft Excel. Each experiment was performed at least three times with 6 replicates for each independent experiment. Data are presented as mean ± standard error of the mean. Differences with *p*-values < 0.05 or <0.05 were considered significant.

### Supplementary information

Supplementary information

Supplementary Table 1

Supplementary Table 2

Supplementary Table 3

Supplementary Table 4

Supplementary Table 5

Supplementary Table 6

nr-reporting-summary

## Data Availability

Sequencing data that support the findings of this study have been deposited in the Gene Expression Omnibus (GEO) under the accession code GSE215980.

## Code availability

Images were analyzed using ZEN (v3.2, Carl Zeiss, Jena, Germany) and ImageJ (v1.53, NIH, Bethesda, MD, USA, https://imagej.nih.gov/ij/). Single-cell data was analyzed with R package Seurat v4. RNA sequencing data was analyzed with R package DESeq2 v1.32. Visualization of bioinformatics results was performed by Seurat v4 and ggplot2 v3. Statistical analysis and data visualization were performed using Microsoft Excel. Custom codes used to generate results reported in the paper are available from the corresponding authors upon reasonable request.
