# Supplementary material for: Epidermal–dermal coupled spheroids are important for tissue pattern regeneration in reconstituted skin explant cultures
Source: NPJ Regen Med. 2023 Nov 23;8:65. doi: 10.1038/s41536-023-00340-0 (PMC10667216; doi:10.1038/s41536-023-00340-0)
Supplement: Supplementary file 1 — Supplementary information [file 41536_2023_340_MOESM1_ESM.pdf]

Supplementary Information for

Epidermal-dermal coupled spheroids are important for tissue pattern regeneration in reconstituted skin explant cultures

Mingxing Lei[1,2]*, Jingwei Jiang[1], Mengyue Wang[1], Wang Wu[1], Jinwei Zhang[1], Wanqian Liu[1], Wei Zhou[3], Yung-Chih Lai[2], Ting-Xin Jiang[4], Randall B Widelitz[4], Hans I Harn[4], Li Yang[1], Cheng-Ming Chuong[4]*

Correspondence: *Mingxing Lei, mingxing@cqu.edu.cn; *Cheng-Ming Chuong, cmchuong@usc.edu

This PDF file includes:

Supplementary Figures 1 to 8

Supplementary Tables 1 to 9

Supplementary References

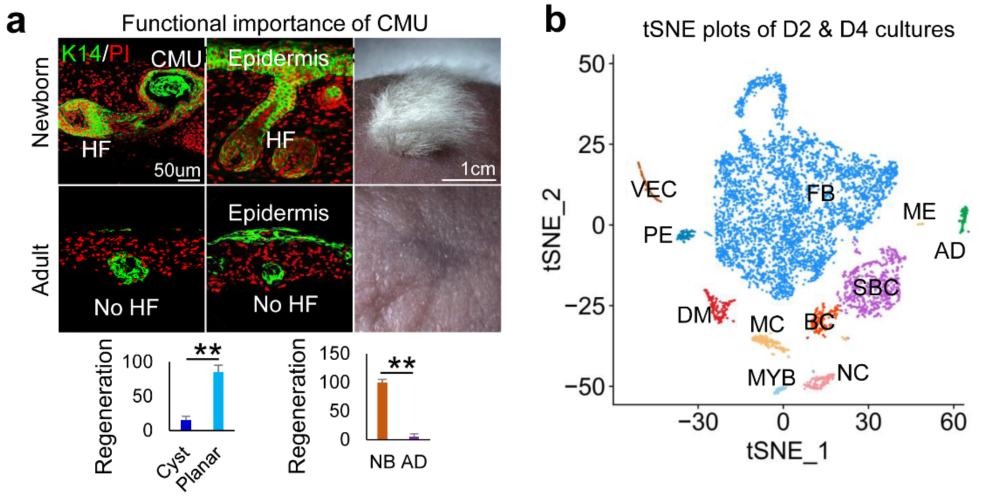

c **Examples of signature genes in epidermal and dermal subclusters**

Clusters	BC	SBC I	SBC II	SBC III	FB I	FB II	FB III	FB IV
Representative signature genes	*Itgb4*	*Fabp5*	*Krt79*	*Klk7*	*Tnc*	*Lamc3*	*Cspg4*	*Car6*
	Col17a1	*BC100530*	*Lurap1l*	*Klk6*	*Thbs2*	*Col6a2*	*Malat1*	*Procr*
	Lamb3	*Krtdap*	*Sox4*	*Sprr1a*	*Mmp2*	*Col6a1*	*Gm47283*	*Manf*
	Lamc2	*Serpinb3b*	*Lmo1*	*Ctsl*	*Efemp2*	*Ifitm1*	*mt-Cytb*	*Derl3*
	Mt2	*Rbp2*	*Ccl27a*	*Cdsn*	*Pcsk5*	*Pdgfrb*	*mt-Atp6*	*Rpl12*
	Fth1	*Ly6d*	*Efna1*	*Sbsn*	*Aebp1*	*Igfbp3*	*Son*	*Slc7a11*
	Slco2a1	*Krt6a*	*Car12*	*Spink5*	*Cpxm1*	*Tmem176a*	*Gm26917*	*Hsp90b1*
	Cxcl14	*Krt16*	*Acot1*	*Asprv1*	*Sod3*	*Crispld2*	*mt-Co2*	*Hspe1*
	Mmp9	*Stfa3*	*Rgcc*	*Crct1*	*Lpl*	*Eln*	*mt-Co3*	*Rplp0*
	Mmp13	*Krt6b*	*Gm26870*	*Cst6*	*Dcn*	*Cbln1*	*Ankrd11*	*Rps5*

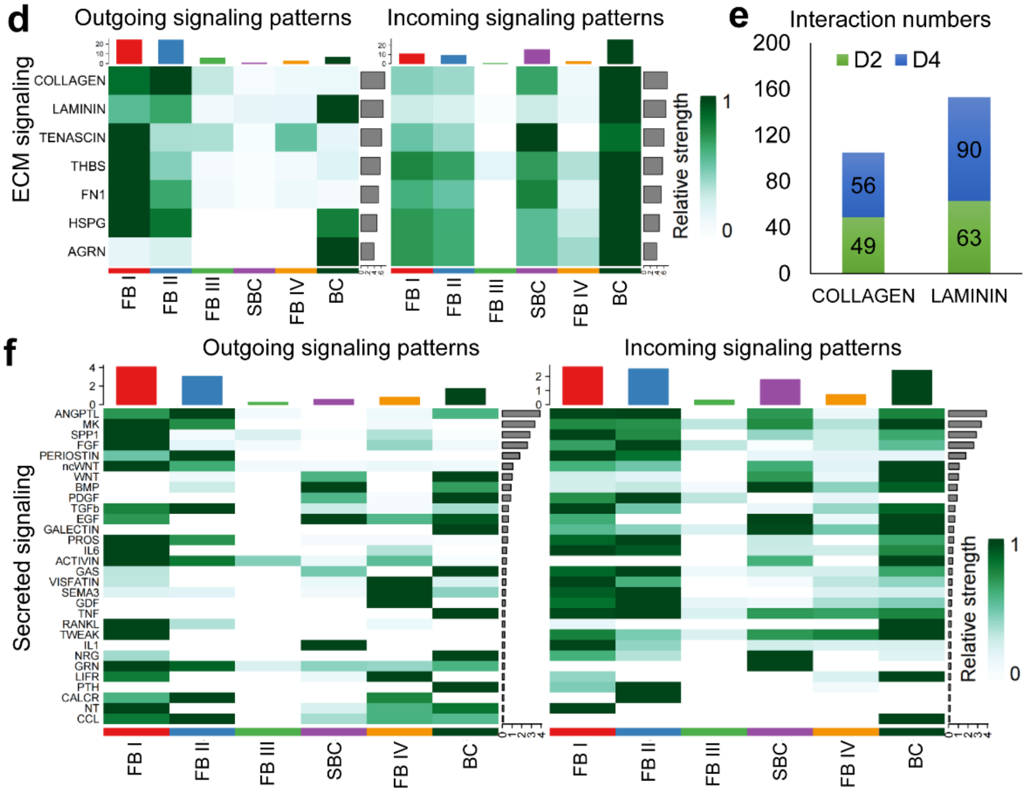

Supplementary Figure 1. Skin cyst formation during newborn mouse skin explant culture.

a. K14 immunostaining shows that hair follicles can be formed at the border of the skin cyst or from a planarized skin upon transplantation with newborn mouse cells-derived skin explant cultures. Lower left bar chart shows the quantification of hair regeneration from cyst or from planarized skin. Lower right bar chart shows the quantification of hair regeneration from newborn (NB) or Adult (AD) cell cultures. **$p<0.01$. $N\geq3$.
b. TSNE plots of combined D2 & D4 cultures. AD, adipocyte; BC, basal cell; FB, fibroblast; DM, dermal macrophage; MC, mast cell; ME, melanocyte; MYB, myoblast; NC, neuro cell; PE, pericyte; SBC, suprabasal cell; VEC, vascular endothelial cell.
c. Examples of signature genes in epidermal and dermal cell clusters. False discovery rate <0.05 and Log2 fold change >1.
d. Heatmap shows the outgoing and incoming signaling patterns by ECM mode. X-axis represents the sender (outgoing) or receiver (incoming) cells, and Y-axis represents the ECM signaling.
e. Bar chart shows increased interaction numbers of Collagen and Laminin between epidermal and dermal cells at D4 vs D2.
f. Heatmap shows the outgoing and incoming signaling patterns by secreted signaling mode. X-axis represents the sender (outgoing) or receiver (incoming) cells, and Y-axis represents the ECM signaling.

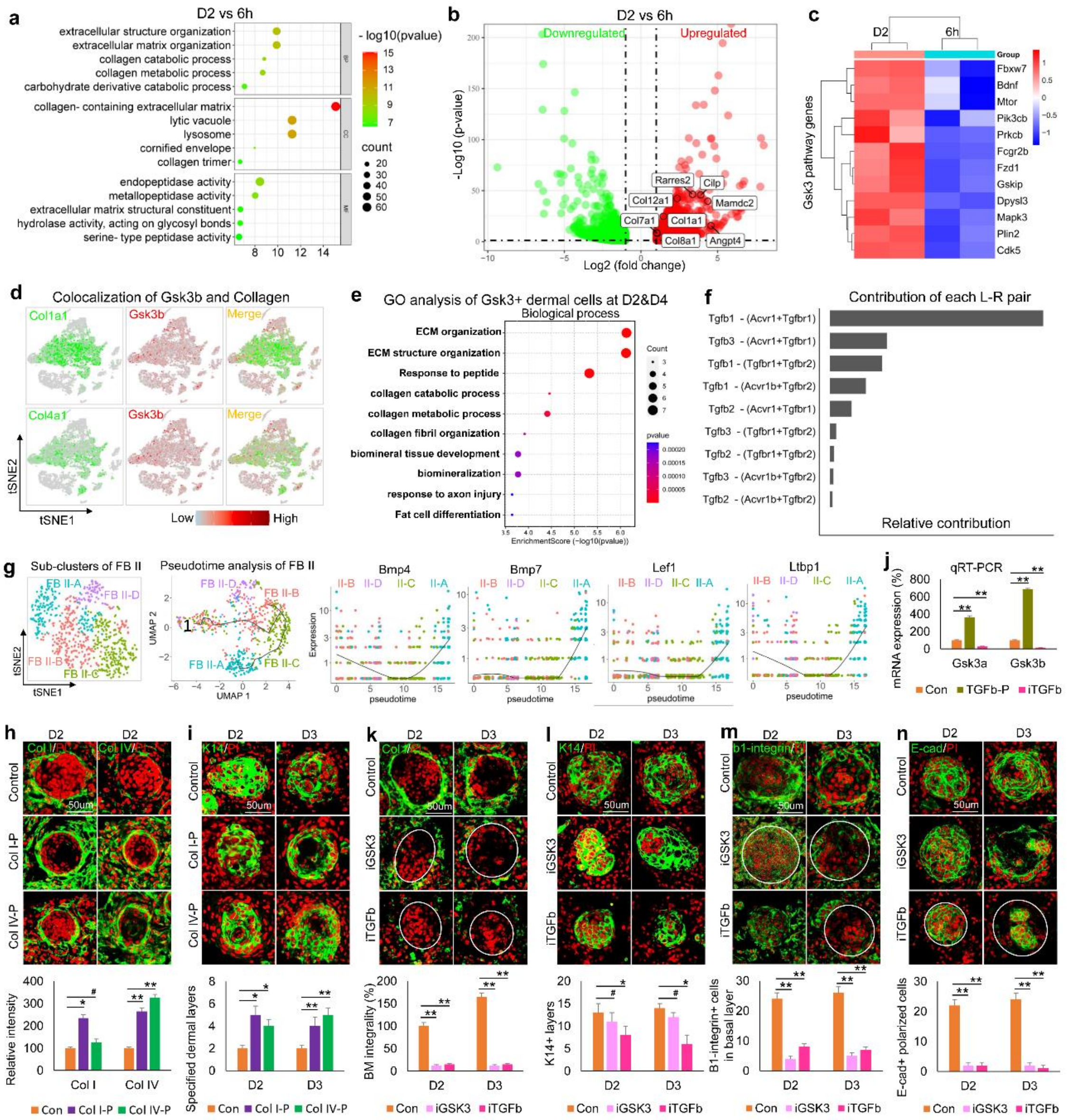

a
D2 vs 6h
extracellular structure organization
extracellular matrix organization
collagen catabolic process
collagen metabolic process
carbohydrate derivative catabolic process
collagen- containing extracellular matrix
lytic vacuole
lysosome
cornified envelope
collagen trimer
endopeptidase activity
metallopeptidase activity
extracellular matrix structural constituent
hydrolase activity, acting on glycosyl bonds
serine- type peptidase activity
- log10(pvalue)
count
b
D2 vs 6h
Downregulated
Upregulated
-Log10 (p-value)
Log2 (fold change)
Rarres2
Cilp
Col12a1
Mamdc2
Col7a1
Col1a1
Col8a1
Angpt4
c
D2
6h
Group
Gsk3 pathway genes
Fbxw7
Bdnf
Mtor
Pik3cb
Prkcb
Fcgr2b
Fzd1
Gskip
Dpysl3
Mapk3
Plin2
Cdk5
d
Colocalization of Gsk3b and Collagen
Col1a1
Gsk3b
Merge
Col4a1
tSNE2
tSNE1
Low
High
e
GO analysis of Gsk3+ dermal cells at D2&D4
Biological process
ECM organization
ECM structure organization
Response to peptide
collagen catabolic process
collagen metabolic process
collagen fibril organization
biomineral tissue development
biomineralization
response to axon injury
Fat cell differentiation
Count
pvalue
EnrichmentScore (-log10(pvalue))
f
Contribution of each L-R pair
Tgfb1 - (Acvr1+Tgfbr1)
Tgfb3 - (Acvr1+Tgfbr1)
Tgfb1 - (Tgfbr1+Tgfbr2)
Tgfb1 - (Acvr1b+Tgfbr2)
Tgfb2 - (Acvr1+Tgfbr1)
Tgfb3 - (Tgfbr1+Tgfbr2)
Tgfb2 - (Tgfbr1+Tgfbr2)
Tgfb3 - (Acvr1b+Tgfbr2)
Tgfb2 - (Acvr1b+Tgfbr2)
Relative contribution
g
Sub-clusters of FB II
FB II-A
FB II-D
FB II-B
FB II-C
Pseudotime analysis of FB II
UMAP 2
UMAP 1
Bmp4
Bmp7
Lef1
Ltbp1
II-B
II-D
II-C
II-A
Expression
pseudotime
j
qRT-PCR
mRNA expression (%)
Gsk3a
Gsk3b
Con
TGFb-P
iTGFb
h
D2
Col I/PI
Col IV/PI
Control
Col I-P
Col IV-P
50um
Relative intensity
Col I
Col IV
Con
Col I-P
Col IV-P
i
D2
D3
K14/PI
Specified dermal layers
k
Col IV/PI
iGSK3
iTGFb
BM integrity (%)
iGSK3
l
K14/PI
K14+ layers
m
b1-integrin/PI
B1-integrin+ cells in basal layer
n
E-cad/PI
E-cad+ polarized cells

Supplementary Figure 2. TGFb-Gsk3 regulates ECM expression in the specified dermal cells in newborn mouse skin explant cultures.

a. Gene ontology of Biological Process (BP), Cellular Components (CC), and Molecular Function (MF) by comparing gene expression between 6h and D2 samples.
b. Volcano plots show upregulated ECM genes in D2 vs 6h cultures. *$p<0.05$, n=2.
c. Heatmap shows Gsk3 pathway gene expression is significantly increased at D2 vs 6h. *$p<0.05$, n=2.
d. T-SNE plots show colocalization of Gsk3b with Col1a1 or Col4a1 in dermal but not in epidermal cells at D2.
e. Gene ontology (GO) and KEGG analysis of Gsk3+ dermal cells in D2 & D4 cultures.
f. Tgfb pathway genes (ligands and receptors) expressed in epidermal and dermal cells.
g. TSNE plots show that dermal papilla marker genes are highly expressed in FB II-A subclusters.
h. Immunostaining and quantification show Collagens I and IV expression at D2 after addition of Collagens I or IV recombinant protein from D0.
i. K14 immunostaining and quantification show increased layers of specified dermal cells at D2 and D3 after addition of Collagens I or IV recombinant protein from D0.
j. qRT-PCR shows increased expression of Gsk3a and Gsk3b at D2 after Tgfb recombinant protein or inhibitor (iTGFb) treatment from D0. **$p<0.01$. N=3.
k. Immunostaining and quantification show that Collagen I expression is decreased at the basement membrane region at D2 and D3 after inhibition of GSK3 or TGFb from D0.

l. K14 immunostaining and quantification show that inhibition of TGFb from D0 leads to smaller aggregate formation at D2 and D3.

m. Immunostaining and quantification show that b1-integrin expression is decreased in the outer layer of the epidermal aggregate at D2 and D3 after inhibition of GSK3 or TGFb from D0.

n. Immunostaining and quantification show decreased E-cadherin+ polarized cells in number at the outer layer of the epidermal aggregate at D2 and D3 after inhibition of Gsk3 or TGFb from D0. *$p<0.05$, **$p<0.01$, and #, no statistical significance. $N \geq 3$.

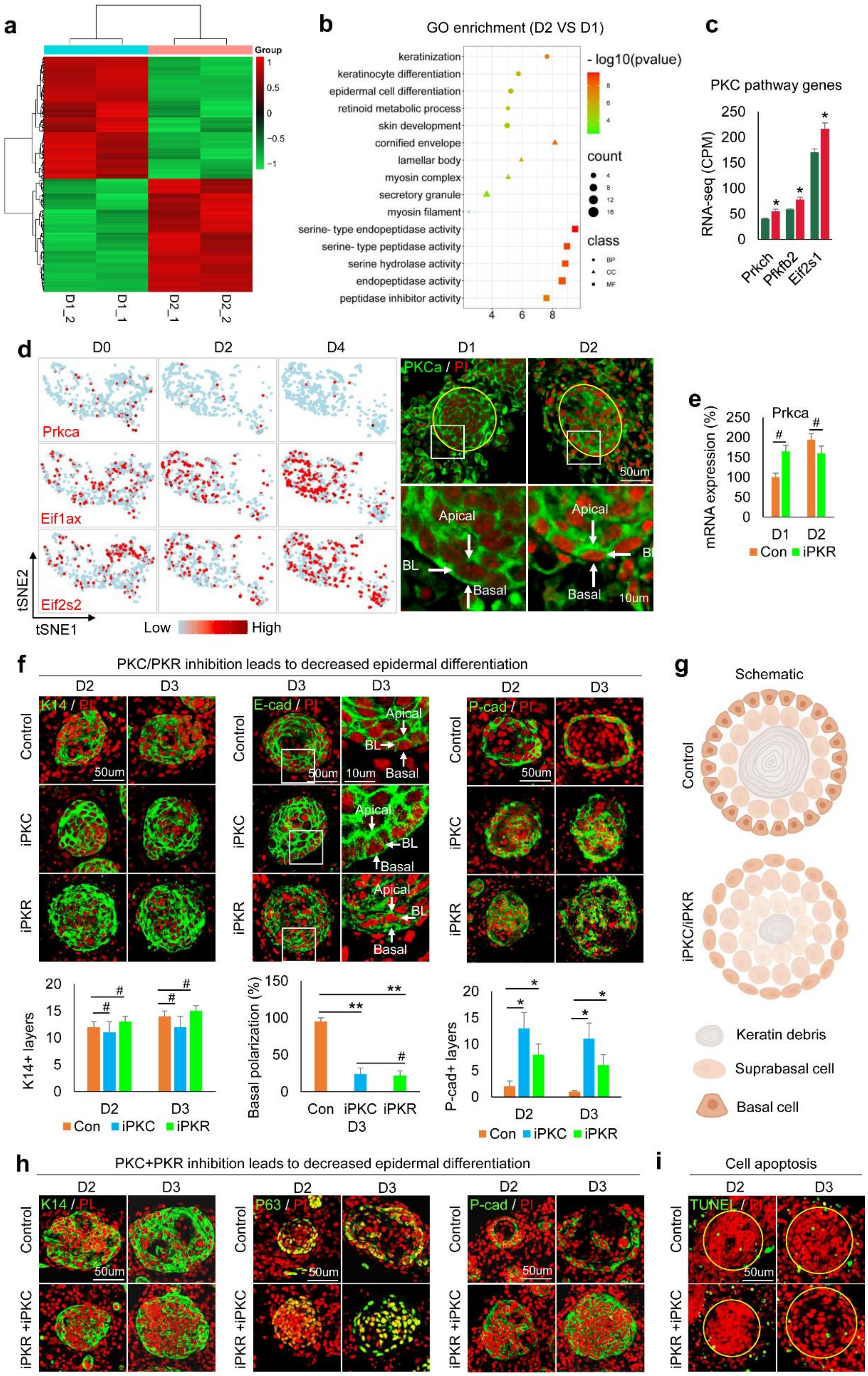

a
Group
D1_2
D1_1
D2_1
D2_2
b
GO enrichment (D2 VS D1)
keratinization
keratinocyte differentiation
epidermal cell differentiation
retinoid metabolic process
skin development
cornified envelope
lamellar body
myosin complex
secretory granule
myosin filament
serine- type endopeptidase activity
serine- type peptidase activity
serine hydrolase activity
endopeptidase activity
peptidase inhibitor activity
- log10(pvalue)
count
class
c
PKC pathway genes
RNA-seq (CPM)
Prkch
Pfkfb2
Eif2s1
d
D0
D2
D4
D1
D2
Prkca
Eif1ax
Eif2s2
tSNE2
tSNE1
Low
High
PKCa / PI
50um
Apical
BL
Basal
10um
e
Prkca
mRNA expression (%)
Con
iPKR
f
PKC/PKR inhibition leads to decreased epidermal differentiation
K14 / PI
E-cad / PI
P-cad / PI
Control
iPKC
iPKR
K14+ layers
Basal polarization (%)
P-cad+ layers
g
Schematic
Control
iPKC/iPKR
Keratin debris
Suprabasal cell
Basal cell
h
PKC+PKR inhibition leads to decreased epidermal differentiation
P63 / PI
iPKR +iPKC
i
Cell apoptosis
TUNEL / PI

Supplementary Figure 3. PKR-PKC promotes epidermal cyst formation in newborn mouse skin explant culture.

a. Hierarchical clustering shows differential gene expression at D1 and D2. *$p<0.05$, $n=2$.
b. Gene ontology of Biological Process (BP), Cellular Components (CC), and Molecular Function (MF) by comparing gene expression in D1 and D2 samples.
c. RNA-seq analysis shows PKC pathway genes upregulated from D1 to D2. *$p<0.05$, $n=2$.
d. Expression of exemplary PKC and PKR pathway genes projected on a tSNE at D0, D2, and D4. Immunostaining verifies PKCa expression in D1 and D2 cultures.
e. qRT-PCR shows that inhibition of PKR doesn't influence Prkca mRNA expression. #, no statistical significance. $N=3$.
f. Immunostaining and quantification of K14 shows that inhibition of PKC (iPKC) or PKR (iPKR) doesn't influence aggregate size at D2 and D3. Immunostaining and quantification of E-cadherin expression after inhibition of PKC or PKR results in delayed apical-basal polarity formation in the basal layer at D2 and D3. Immunostaining and quantification of basal cell marker (P-cadherin) shows inhibition of PKC or PKR leads to decreased polarization at D2 and D3. *$p<0.05$, **$p<0.01$, and #, no statistical significance. $N\geq3$.
g. Schematic of PKR and PKC in regulates apical-basal polarity formation.
h. Inhibition of PKR and PKC leads to decreased epidermal differentiation.
i. TUNEL staining shows cell apoptosis after inhibition of PKR and PKC at D2 and D3.

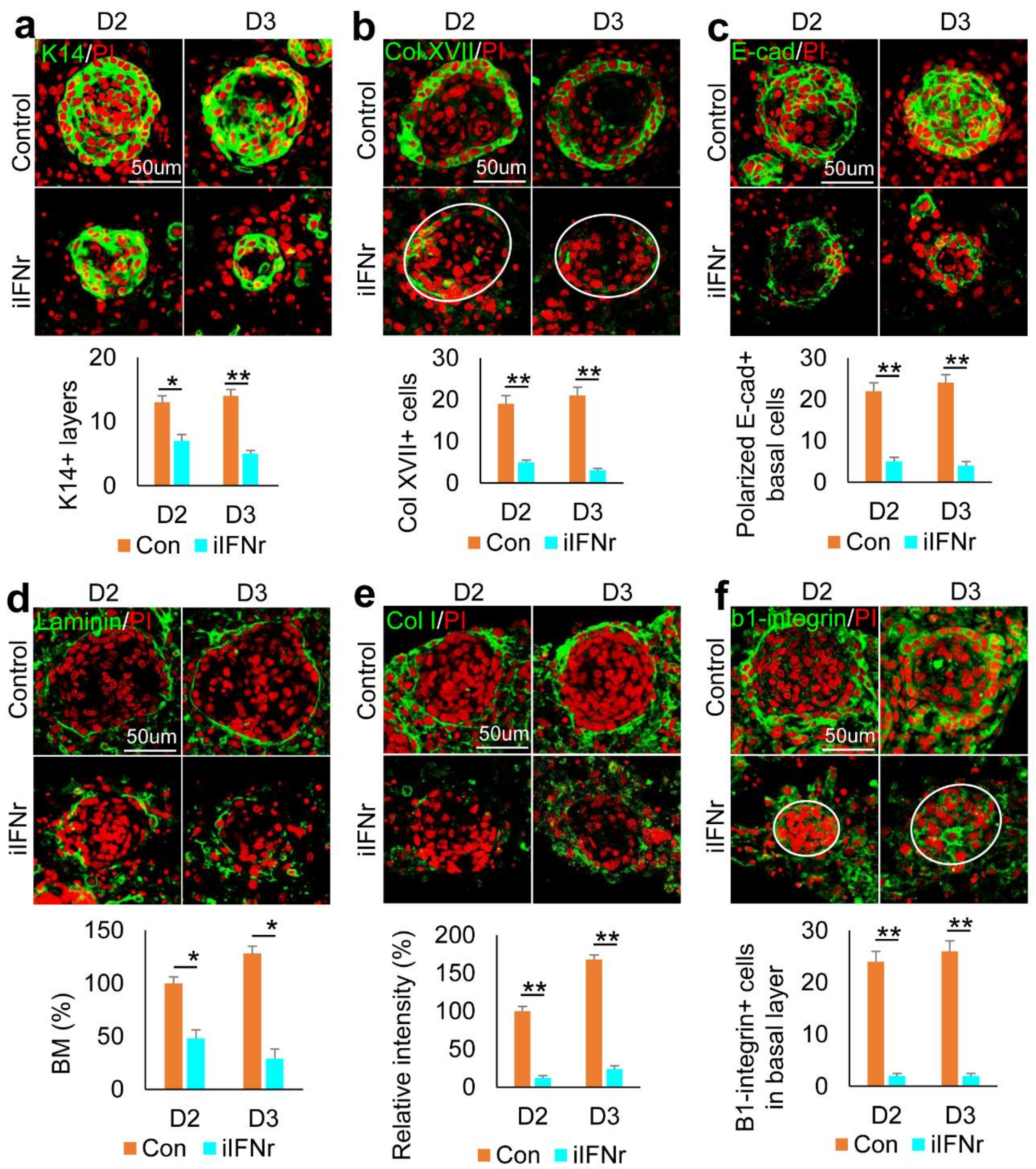

a
D2
D3
K14/PI
Control
iIFNr
50um
20
10
0
K14+ layers
*
**
D2
D3
Con
iIFNr
b
Col XVII/PI
30
20
10
Col XVII+ cells
c
E-cad/PI
Polarized E-cad+ basal cells
d
Laminin/PI
150
100
50
BM (%)
e
Col I/PI
200
150
Relative intensity (%)
f
b1-integrin/PI
B1-integrin+ cells in basal layer

Supplementary Figure 4. **IFNr expression and function in skin cyst of newborn mouse skin explant culture.**

a. Immunostaining and quantification show that inhibition of IFNr leads to smaller aggregate formation at D2, D3 and D4. $^*p<0.05$, $^{**}p<0.01$. N≥3.
b. Immunostaining and quantification show that inhibition of IFNr results in decreased Collagen XVII expression at D2 & D3. $^{**}p<0.01$. N≥3.
c. Immunostaining and quantification show that inhibition of IFNr results in a decrease of polarized E-cadherin expression at D2 & D3. $^{**}p<0.01$. N≥3.
d. Immunostaining and quantification show that inhibition of IFNr results in a disrupted basement membrane (Laminin+) formation at D2 & D3. $^{**}p<0.01$. N≥3.
e. Immunostaining and quantification show that inhibition of IFNr results in a disrupted basement membrane formation (Collagen I+) at D2 & D3. $^*p<0.05$. N≥3.
f. Immunostaining and quantification show that inhibition of IFNr results in decreased b1-integrin expression in the basal layer of the epidermal aggregate at D2 & D3. $^{**}p<0.01$. N≥3.

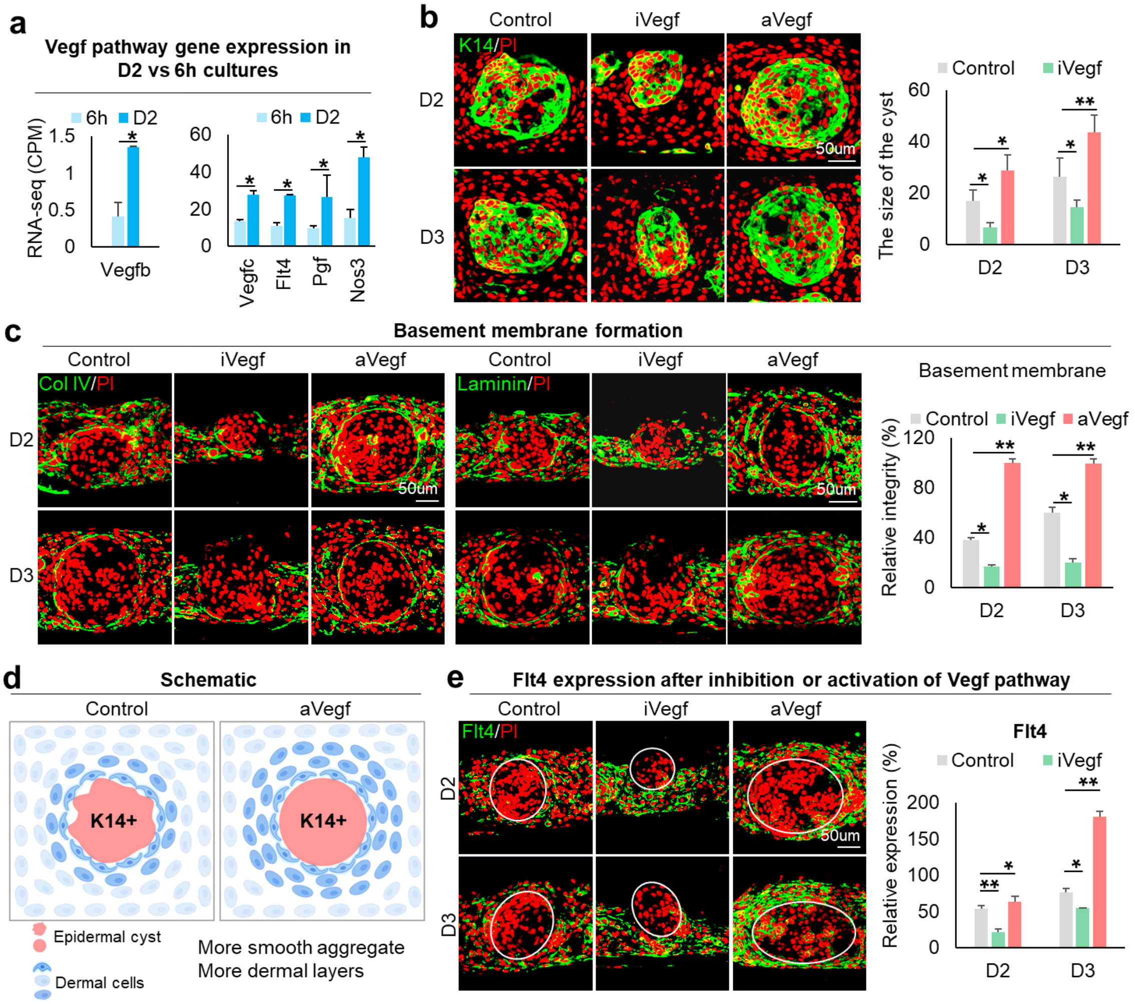
a
Vegf pathway gene expression in D2 vs 6h cultures
6h D2
RNA-seq (CPM)
Vegfb
Vegfc
Flt4
Pgf
Nos3
b
Control
iVegf
aVegf
K14/PI
D2
D3
50um
The size of the cyst
c
Basement membrane formation
Col IV/PI
Laminin/PI
Basement membrane
Relative integrity (%)
d
Schematic
K14+
Epidermal cyst
Dermal cells
More smooth aggregate
More dermal layers
e
Flt4 expression after inhibition or activation of Vegf pathway
Flt4/PI
Flt4
Relative expression (%)

Supplementary Figure 5. **Vegf pathway influences dermal cell attachment to the epidermal aggregate in newborn mouse skin explant culture.**

a. RNA-seq analysis shows increased expression of Vegf pathway genes in D2 vs 6h cultures. $^{*}p<0.05$. N=2.
b. K14 immunostaining and quantification show CMU morphology in Vegf inhibition (iVegf) or Vegf activation (aVegf) groups at D2 & D3. $^{*}p<0.05$, $^{**}p<0.01$. $N\geq3$.
c. Immunostaining for Col IV and Laminin and quantification show basement membrane formation in iVegf or aVegf groups at D2 & D3. $^{*}p<0.05$, $^{**}p<0.01$. $N\geq3$.
d. Schematic of aVegf leads to more dermal cell attachment and smoother aggregate in morphology.
e. Immunostaining and quantification show increased or decreased Flt4 expression in iVegf or aVegf groups at D2 & D3. $^{*}p<0.05$, $^{**}p<0.01$. $N\geq3$.

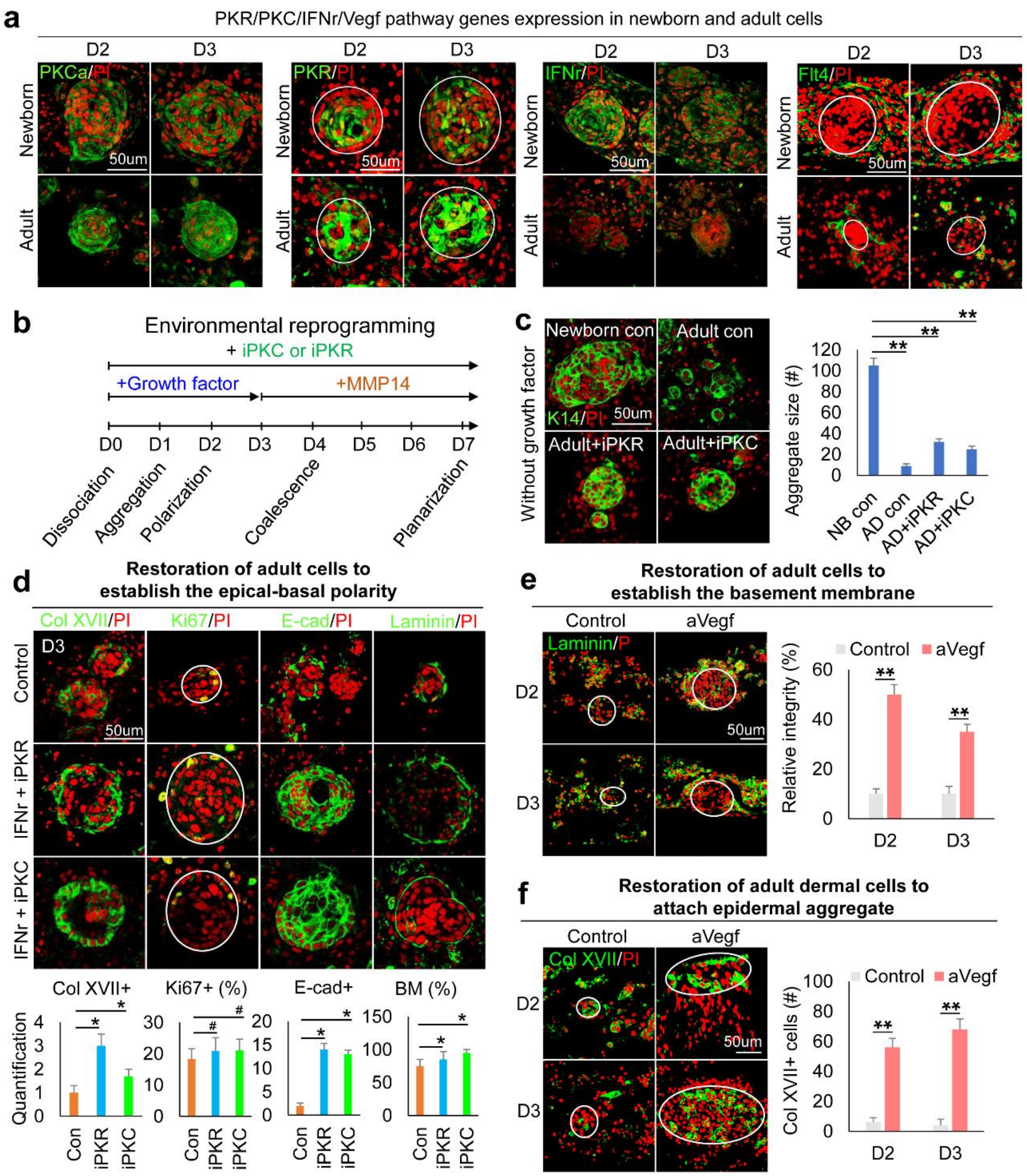
a
PKR/PKC/IFNr/Vegf pathway genes expression in newborn and adult cells
D2
D3
PKCa/PI
PKR/PI
IFNr/PI
Flt4/PI
Newborn
Adult
50um
b
Environmental reprogramming
+ iPKC or iPKR
+Growth factor
+MMP14
D0 D1 D2 D3 D4 D5 D6 D7
Dissociation
Aggregation
Polarization
Coalescence
Planarization
c
Without growth factor
Newborn con
Adult con
K14/PI
Adult+iPKR
Adult+iPKC
Aggregate size (#)
NB con
AD con
AD+iPKR
AD+iPKC
d
Restoration of adult cells to establish the epical-basal polarity
Col XVII/PI
Ki67/PI
E-cad/PI
Laminin/PI
Control
IFNr + iPKR
IFNr + iPKC
Col XVII+
Ki67+ (%)
E-cad+
BM (%)
Quantification
Con
iPKR
iPKC
e
Restoration of adult cells to establish the basement membrane
Control
aVegf
Laminin/P
Relative integrity (%)
f
Restoration of adult dermal cells to attach epidermal aggregate
Col XVII/PI
Col XVII+ cells (#)

Supplementary Figure 6. **Restoration of adult mouse cells to form CMU by environmental reprogramming.**

a. Immunostaining shows PKCa, PKR, IFNr, and VEGF pathway gene expression in newborn and adult cell cultures at D2 and D3.
b. Optimized environmental reprogramming protocol shows delivery of key molecules at different stages in adult cell explant culture.
c. K14 immunostaining and quantification show skin cyst formation in newborn, adult, and iPKR- or iPKC-treated cells. ** $p<0.01$. N≥3.
d. Immunostainings and quantifications of IFNr+iPKR- or IFNr+iPKC-treated adult mouse cells. * $p<0.05$ and #, no statistical significance. N≥3.
e. Immunostaining for Laminin shows basement membrane formation after addition of VEGF recombinant protein. ** $p<0.01$. N≥3.
f. Immunostaining shows that more dermal cells attach to the epidermal aggregate in restored group which show more Col XVII+ epidermal cells. ** $p<0.01$. N≥3.

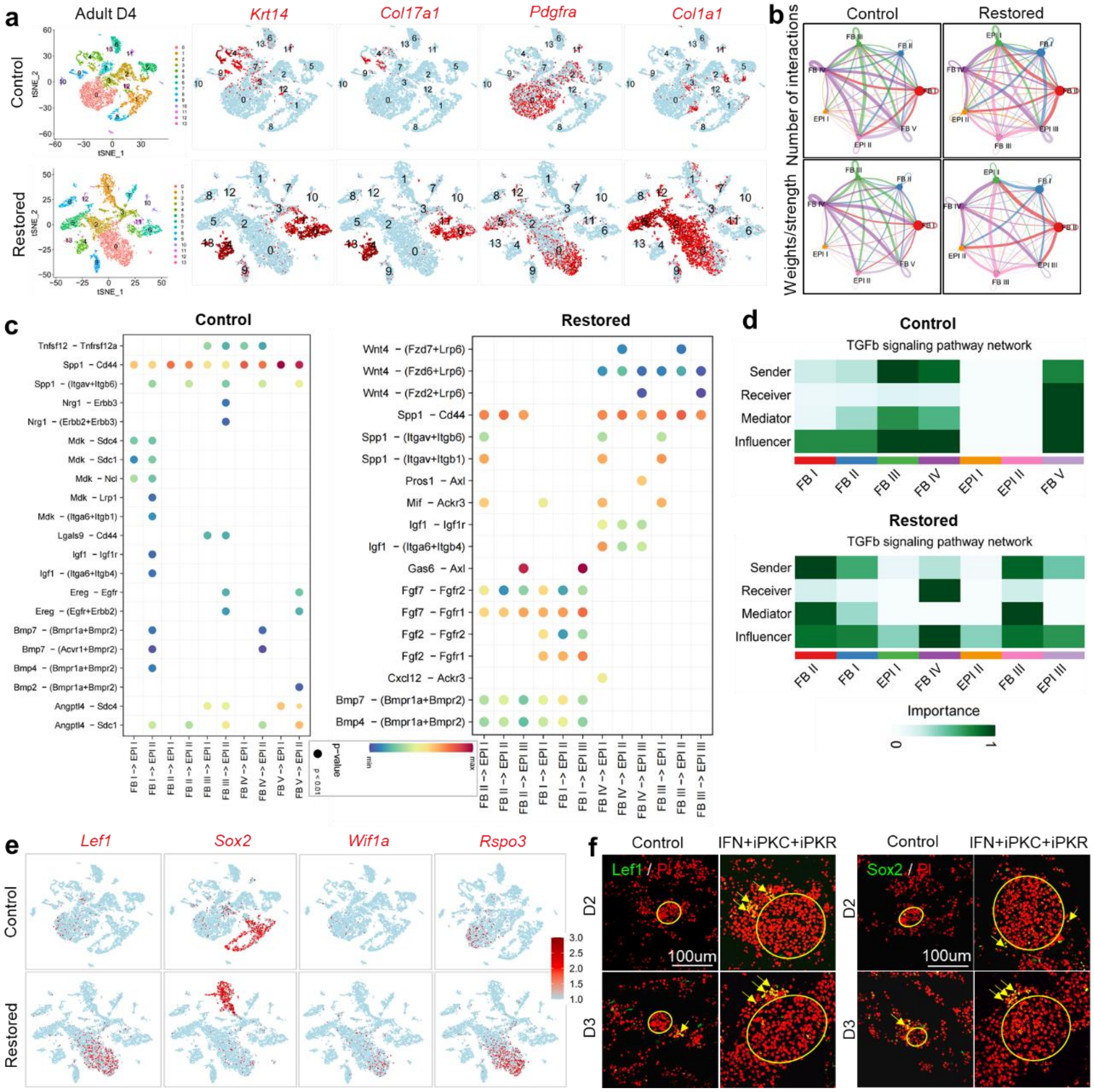
a
Adult D4
Krt14
Col17a1
Pdgfra
Col1a1
Control
Restored
tSNE_1
tSNE_2
b
Control
Restored
Number of interactions
Weights/strength
c
Control
Restored
d
Control
TGFb signaling pathway network
Sender
Receiver
Mediator
Influencer
Restored
TGFb signaling pathway network
Importance
e
Lef1
Sox2
Wif1a
Rspo3
f
Control
IFN+iPKC+iPKR
Lef1 / PI
Sox2 / PI
D2
D3
100um

Supplementary Figure 7. ScRNA-seq analysis of adult mouse cell cultures after environmental reprogramming.

a. TSNE plots show cell clusters and marker genes for epidermal and dermal cells in D4 restored adult cells.
b. Increased number of interactions and enhanced weights/strength between epidermal and dermal cells in D4 restored vs control adult cells.
c. The significant ligand-receptor pairs that contribute to the signaling sending from fibroblasts to epidermal cells show increased EMI in D4 restored vs control adult cells. False discovery rate <0.05 and Log2 fold change >1.
d. Secreted mode of CellChat analysis shows enhanced Tgfb signaling network between epidermal and dermal cells in D4 restored vs control cells.
e. ScRNA-seq analysis shows representative DP marker genes expression in D4 adult cell cultures.
f. Immunostaining shows Lef1 and Sox2 expression in D2 and D3 restored adult mouse cell explant cultures.

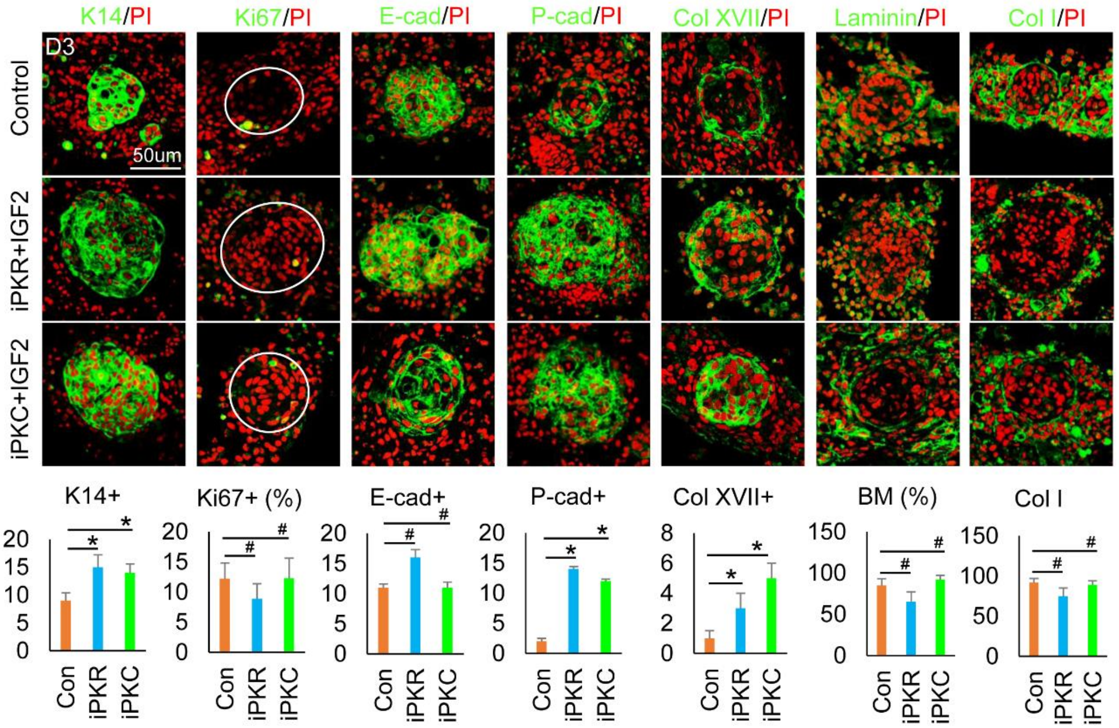

Supplementary Figure 8. Environmental reprograming of human fetal scalp cells to form CMU.

Immunostaining and quantification of K14 shows that inhibition of PKC (iPKC) or PKR (iPKR) doesn’t influence aggregate size at D2 and D3. Immunostaining and quantification of E-cadherin expression after inhibition of PKC or PKR results in delayed apical-basal polarity formation in the basal layer at D2 and D3. Immunostaining and quantification of basal cell marker (P-cadherin, Col XVII) shows inhibition of PKC or PKR leads to decreased polarization at D2 and D3. Immunostaining and quantification show basement membrane formation after inhibition of PKC or PKR. *p<0.05, # no significance. N⩾3.

Supplementary Tables

Supplementary Table 1. All marker genes of sub-clusters from newborn mice skin explant cultures (D2 and D4).

Supplementary Table 2. Interactions of ligands-receptors between different cell clusters in newborn mice skin explant cultures (D2 and D4).

Supplementary Table 3. Differentially expressed genes from newborn mice skin explant cultures (D2 compared to D1).

Supplementary Table 4. Differentially expressed genes from PKR+ cells compared to PKR- cells at D2 newborn mice skin explant cultures.

Supplementary Table 5. All marker genes of sub-clusters from adult mice skin explant cultures.

Supplementary Table 6. Interactions of ligands-receptors between different cell clusters in adult mice skin explant cultures (control and restored).

Supplementary Table 7. Antibodies used in this study.

Antibody	Host/Isotype	Dilution	Company	Cat #
b-catenin	Mouse	1:200	BD Biosciences	610153
b1-integrin	Rabbit	1:200	Genetex	GTX128839
Collagen I	Goat	1:500	Arigobio	ARG21965
Collagen IV	Rabbit	1:500	Abcam	ab19808
Collagen XVII	Rabbit	1:500	Abcam	ab184996
E-cadherin	Mouse	1:200	BD Biosciences	610181

FABP5	Rabbit	1:200	Proteintech	12348-1-AP
Gsk3 beta	Rabbit	1:200	Beyotime	AF1543
IFNr	Mouse	1:200	Biolegend	505801
IFNGR2	Rabbit	1:200	ABSCI	AB37643
Klk6	Goat	1:500	Thermo	PA5-47239
Klk7	Goat	1:500	R&D systems	AF2624
Krt14	Mouse	1:500	Thermo	MA5-11599
Laminin	Rabbit	1:500	Abcam	ab11575
Lamp2	Mouse	1:100	Santa Cruz	sc-20004
P63	Rabbit	1:200	Proteintech	12143-1-AP
P-cadherin	Goat	1:200	R&D systems	AF761
Pdgfra	Rat	1:100	eBioscience	14-1401-82
PKCα	Rabbit	1:100	Proteintech	21991-1-AP
PKCδ	Rabbit	1:100	Elabscience	E-AB-32605
PKCζ	Rabbit	1:100	Elabscience	E-AB-32609
PKR	Rabbit	1:100	Proteintech	18244-1-AP
p-Stat3	Mouse	1:200	Cell signaling	9136
Smad1	Rabbit	1:100	Beyotime	AF1504
Stat1	Rabbit	1:200	Cell signaling	14994s
Tgfbr3	Rabbit	1:100	Bioss	Bs-1910R
Vimentin	Mouse	1:100	Santa Cruz	sc-373717
Alexa Fluor488 goat anti-mouse IgG（H+L)	Mouse	1:500	Thermo	A11001

Alexa Fluor488 goat anti rabbit IgG （H+L)	Rabbit	1:500	Thermo	A11008
AF488 donkey anti goat IgG（H+L)	Goat	1:500	Thermo	A11055

Supplementary Table 8. Probes and primers used in this study.

Gene	Primer (5' to 3')
Ifi202b-AS	CTAATACGACTCACTATAGGGcactgtggcatggaacatct
Ifi202b-S	tcaacagcagtggcatccta
Tgfbi-AS	CTAATACGACTCACTATAGGGgagtctgccagctcatctcc
Tgfbi-S	tgataagaggggacggtttg
Prkca-AS	GGGCGATGAATTTGTGGTCTT
Prkca-S	GTTTACCCGGCCAACGACT
Eif2ak2-AS	TGACAATCCACCTTGTTTTCGT
Eif2ak2-S	ATGCACGGAGTAGCCATTACG
Col1a1-AS	CCACGTCTCACCATTGGGG
Col1a1-S	GCTCCTCTTAGGGGCCACT
Col4a1-AS	ACGTGGCCGAGAATTTCACC
Col4a1-S	CTGGCACAAAAGGGACGAG
Gsk3a-AS	GGTCCAGCTTACGCATAATCTG
Gsk3a-S	GCGTTCCCAAGAAGTGGCTTA
Gsk3b-AS	TGGCAGCAAGGTAACCACAG
Gsk3b-S	CGGTTCTTAAATCGCTTGTCCTG

Supplementary Table 9. Small molecules and recombinant protein**.**

Name	Company	Catalog Number	Concentration
Bisindolyl maleimide I (iPKC)	Cayman Chemical	XR-ICAY13298-5MG	5 uM

Chelerythrine Chloride (iPKC)	Cayman Chemical	3892-92-9	5 uM
SB 431542 hydrate (iTgfb)	Sigma	S4317	10 uM
PKR inhibitor	Sigma	I9785	5 uM
SAR-20347	Biovision	B1290-5mg	10 uM
Chir99021, Gsk3 inhibitor	Sigma	SML1046	5 uM
IGF2 recombinant protein	Peprotech	PEP100-12-10	10 ng/ml
Collagen I recombinant protein	Thermo	A1048301	20 ng/ml
Collagen IV recombinant protein	Sigma	C6745	20 ng/ml
MMP14 Recombinant Protein	Aviva systems biology	OPCD05324	10 ng/ml
Tgfb recombinant protein	Thermo	PHG9214	20 ng/ml
Interferon-γ recombinant protein	Sigma	IF005	20 ng/ml

Supplementary References

1. Lim, C. H. et al. Hedgehog stimulates hair follicle neogenesis by creating inductive dermis during murine skin wound healing. Nat Commun 9, 4903, doi:10.1038/s41467-018-07142-9 (2018).

2. Harn, H. I. et al. Symmetry breaking of tissue mechanics in wound induced hair follicle regeneration of laboratory and spiny mice. Nat Commun 12, 2595, doi:10.1038/s41467-021-22822-9 (2021).

3. Phan, Q. M. et al. Lef1 expression in fibroblasts maintains developmental potential in adult skin to regenerate wounds. Elife 9, doi:10.7554/eLife.60066 (2020).

4. Gay, D. et al. Fgf9 from dermal gammadelta T cells induces hair follicle neogenesis after wounding. Nat Med 19, 916-923, doi:10.1038/nm.3181 (2013).

5. Kim, D. et al. Noncoding dsRNA induces retinoic acid synthesis to stimulate hair follicle regeneration via TLR3. Nat Commun 10, 2811, doi:10.1038/s41467-019-10811-y (2019).

6. Collier, A. et al. Gibbin mesodermal regulation patterns epithelial development. Nature 606, 188-196, doi:10.1038/s41586-022-04727-9 (2022).

7. Driskell, R. R. et al. Distinct fibroblast lineages determine dermal architecture in skin development and repair. Nature 504, 277-281, doi:10.1038/nature12783 (2013).

8. Sick, S., Reinker, S., Timmer, J. & Schlake, T. WNT and DKK determine hair follicle spacing through a reaction-diffusion mechanism. Science 314, 1447-1450, doi:10.1126/science.1130088 (2006).

9. Huh, S. H. et al. Fgf20 governs formation of primary and secondary dermal condensations in developing hair follicles. Genes Dev 27, 450-458, doi:10.1101/gad.198945.112 (2013).

10. Biggs, L. C. et al. Hair follicle dermal condensation forms via Fgf20 primed cell cycle exit, cell motility, and aggregation. Elife 7, doi:10.7554/eLife.36468 (2018).

11. Jung, H. S. et al. Local inhibitory action of BMPs and their relationships with activators in feather formation: implications for periodic patterning. Dev Biol 196, 11-23, doi:10.1006/dbio.1998.8850 (1998).
12. Zhang, Y. et al. Reciprocal requirements for EDA/EDAR/NF-kappaB and Wnt/beta-catenin signaling pathways in hair follicle induction. Dev Cell 17, 49-61, doi:10.1016/j.devcel.2009.05.011 (2009).
13. Chiang, C. et al. Essential role for Sonic hedgehog during hair follicle morphogenesis. Dev Biol 205, 1-9, doi:10.1006/dbio.1998.9103 (1999).
14. Gupta, K. et al. Single-Cell Analysis Reveals a Hair Follicle Dermal Niche Molecular Differentiation Trajectory that Begins Prior to Morphogenesis. Dev Cell 48, 17-31 e16, doi:10.1016/j.devcel.2018.11.032 (2019).
15. Fliniaux, I., Mikkola, M. L., Lefebvre, S. & Thesleff, I. Identification of dkk4 as a target of Eda-A1/Edar pathway reveals an unexpected role of ectodysplasin as inhibitor of Wnt signalling in ectodermal placodes. Dev Biol 320, 60-71, doi:10.1016/j.ydbio.2008.04.023 (2008).
