# Supplementary material for: Epidermal–dermal coupled spheroids are important for tissue pattern regeneration in reconstituted skin explant cultures
Source: NPJ Regen Med. 2023 Nov 23;8:65. doi: 10.1038/s41536-023-00340-0 (PMC10667216; doi:10.1038/s41536-023-00340-0)
Supplement: Supplementary file 8 — nr-reporting-summary [file 41536_2023_340_MOESM8_ESM.pdf]

nature portfolio

Corresponding author(s): Mingxing Lei�Cheng-Ming Chuong

Last updated by author(s): May 11, 2023

Reporting Summary

Nature Portfolio wishes to improve the reproducibility of the work that we publish. This form provides structure for consistency and transparency in reporting. For further information on Nature Portfolio policies, see our Editorial Policies and the Editorial Policy Checklist.

Statistics

For all statistical analyses, confirm that the following items are present in the figure legend, table legend, main text, or Methods section.

n/a	Confirmed	
[ ]	[x]	The exact sample size (*n*) for each experimental group/condition, given as a discrete number and unit of measurement
[ ]	[x]	A statement on whether measurements were taken from distinct samples or whether the same sample was measured repeatedly
[ ]	[x]	The statistical test(s) used AND whether they are one- or two-sided<br>*Only common tests should be described solely by name; describe more complex techniques in the Methods section.*
[ ]	[x]	A description of all covariates tested
[ ]	[x]	A description of any assumptions or corrections, such as tests of normality and adjustment for multiple comparisons
[ ]	[x]	A full description of the statistical parameters including central tendency (e.g. means) or other basic estimates (e.g. regression coefficient) AND variation (e.g. standard deviation) or associated estimates of uncertainty (e.g. confidence intervals)
[ ]	[x]	For null hypothesis testing, the test statistic (e.g. *F*, *t*, *r*) with confidence intervals, effect sizes, degrees of freedom and *P* value noted<br>*Give P values as exact values whenever suitable.*
[x]	[ ]	For Bayesian analysis, information on the choice of priors and Markov chain Monte Carlo settings
[x]	[ ]	For hierarchical and complex designs, identification of the appropriate level for tests and full reporting of outcomes
[x]	[ ]	Estimates of effect sizes (e.g. Cohen's *d*, Pearson's *r*), indicating how they were calculated

Our web collection on statistics for biologists contains articles on many of the points above.

Software and code

Policy information about availability of computer code

Data collection	no software were used.
Data analysis	The D0, D2, and D4 newborn mice skin organoid cultures were collected for single-cell RNA sequencing. Seurat v 4.1.2 was used for quality control, dimensionality reduction, and clustering. For each sample dataset, we filtered the expression matrix by the following criteria: 1) cells with gene count less than 500 or with top 2% gene count were excluded; 2) cells with top 2% unique molecular identifiers (UMIs) count were excluded; 3) cells with mitochondrial content > 5% were excluded; 4) genes expressed in less than 5 cells were excluded. The top 20 principal components were selected for tSNE and UMAP reduction. The marker genes of each cluster were identified by the seurat functions FindNeighbours and FindClusters. The single R package and Cell Marker online database were used for the definition of each cell cluster. Visualization of genes was performed by seurat v4 and ggplot2 v3. D0 or D4 epidermal cells clusters (suprabasal cells and basal cells) or dermal cells were selected for pseudotime analysis. The R package monocle v3 was used for establishing the lineage trajectory of different cell clusters. After the annotation of cell clusters, four groups of dermal cells (FBI, FB II, FB III, FB IV) and two groups of epidermal cells (BC, SBC) were selected for cell communication analysis. Representative signature genes were used to annotate epidermal and dermal subclusters. CellChat R package v1.1.3 was used to analyze the ligand-receptor interaction. “ECM-receptor” and “Secreted signaling” modes were selected for the epidermal-dermal interaction analysis

For manuscripts utilizing custom algorithms or software that are central to the research but not yet described in published literature, software must be made available to editors and reviewers. We strongly encourage code deposition in a community repository (e.g. GitHub). See the Nature Portfolio guidelines for submitting code & software for further information.

Data

Policy information about availability of data

All manuscripts must include a data availability statement. This statement should provide the following information, where applicable:

- Accession codes, unique identifiers, or web links for publicly available datasets
- A description of any restrictions on data availability
- For clinical datasets or third party data, please ensure that the statement adheres to our policy

Sequencing data that support the findings of this study have been deposited in the Gene Expression Omnibus (GEO) under the accession code GSE215980.

Research involving human participants, their data, or biological material

Policy information about studies with human participants or human data. See also policy information about sex, gender (identity/presentation), and sexual orientation and race, ethnicity and racism.

Reporting on sex and gender	Not applicable
Reporting on race, ethnicity, or other socially relevant groupings	Not applicable
Population characteristics	Not applicable
Recruitment	Not applicable
Ethics oversight	Not applicable

Note that full information on the approval of the study protocol must also be provided in the manuscript.

Field-specific reporting

Please select the one below that is the best fit for your research. If you are not sure, read the appropriate sections before making your selection.

☒ Life sciences ☐ Behavioural & social sciences ☐ Ecological, evolutionary & environmental sciences

For a reference copy of the document with all sections, see nature.com/documents/nr-reporting-summary-flat.pdf

Life sciences study design

All studies must disclose on these points even when the disclosure is negative.

Sample size	No statistical methods were used to predetermine sample size
Data exclusions	No data were excluded from the analyses.
Replication	Data were pooled for two-three biologically independent experiments as mentioned in figure legends and methods section on Statistics and Reproducibility
Randomization	Samples were allocated randomly for imaging and analyses. There was no bias in selecting particular cells within an image
Blinding	Not applicable

Reporting for specific materials, systems and methods

We require information from authors about some types of materials, experimental systems and methods used in many studies. Here, indicate whether each material, system or method listed is relevant to your study. If you are not sure if a list item applies to your research, read the appropriate section before selecting a response.

Materials & experimental systems

n/a	Involved in the study
☐	☒ Antibodies
☒	☐ Eukaryotic cell lines
☒	☐ Palaeontology and archaeology
☐	☒ Animals and other organisms
☒	☐ Clinical data
☒	☐ Dual use research of concern
☒	☐ Plants

Methods

n/a	Involved in the study
☒	☐ ChIP-seq
☒	☐ Flow cytometry
☒	☐ MRI-based neuroimaging

Antibodies

Antibodies used

Antibody Host/Isotype Company Cat #
b-catenin Mouse BD Biosciences 610153
b1-integrin Rabbit Genetex GTX128839
Collagen I Goat Arigobio ARG21965
Collagen IV Rabbit Abcam ab19808
Collagen XVII Rabbit Abcam ab184996
E-cadherin Mouse BD Biosciences 610181
FABP5 Rabbit Proteintech 12348-1-AP
Gsk3 beta Rabbit Beyotime AF1543
IFNr Mouse Biolegend 505801
IFNGR2 Rabbit ABSCI AB37643
Klk6 Goat Thermo PA5-47239
Klk7 Goat R&D systems AF2624
Krt14 Mouse Thermo MA5-11599
Laminin Rabbit Abcam ab11575
Lamp2 Mouse Santa Cruz sc-20004
P63 Rabbit Proteintech 12143-1-AP
P-cadherin Goat R&D systems AF761
Pdgfra Rat eBioscience 14-1401-82
PKCα Rabbit Proteintech 21991-1-AP
PKCδ Rabbit Elabscience E-AB-32605
PKCζ Rabbit Elabscience E-AB-32609
PKR Rabbit Proteintech 18244-1-AP
p-Stat3 Mouse Cell signaling 9136
Smad1 Rabbit Beyotime AF1504
Stat1 Rabbit Cell signaling 14994s
Tgfbr3 Rabbit Bioss Bs-1910R
Vimentin Mouse Santa Cruz sc-373717
Alexa Fluor488 goat anti-mouse IgG�H+L) Mouse Thermo A11001
Alexa Fluor488 goat anti rabbit IgG�H+L) Rabbit Thermo A11008
AF488 donkey anti goat IgG�H+L) Goat Thermo A11055

Validation

such as, Collagen I, https://www.arigobio.cn/anti-Collagen-I-antibody-pre-adsorbed-ARG21965.html#tabs-publications

Animals and other research organisms

Policy information about studies involving animals; ARRIVE guidelines recommended for reporting animal research, and Sex and Gender in Research

Laboratory animals

K14H2BGFP mice, CD1 and nude mice

Wild animals

No

Reporting on sex

No

Field-collected samples

Mice were kept at 26? with a 12-h light cycle, with food and water ad libitum

Ethics oversight

Institutional Animal Care and Use Committees of Chongqing University

Note that full information on the approval of the study protocol must also be provided in the manuscript.
